# Oral Antidiabetic Agents: From Routine Quality Control to Bioanalysis

**DOI:** 10.3390/molecules31162915

**Published:** 2026-08-20

**Authors:** Ana-Maria Toma, Larisa Păduraru, Romeo Petru Dobrin, Nela Bibire, Mădălina Vieriu, Mihai Apostu

**Affiliations:** 1 Faculty of Pharmacy, Department of Analytical Chemistry, Grigore T. Popa University of Medicine and Pharmacy Iasi, 16th Universitatii Street, 700116 Iasi, Romania; ana-maria.toma@d.umfiasi.ro (A.-M.T.); nela.bibire@umfiasi.ro (N.B.); madalina.vieriu@umfiasi.ro (M.V.); mihai.apostu@umfiasi.ro (M.A.); 2 Faculty of Medicine, Department of Medicine III, Grigore T. Popa University of Medicine and Pharmacy Iasi, 16th Universitatii Street, 700116 Iasi, Romania; petru.dobrin@umfiasi.ro

**Keywords:** oral antidiabetic agents, instrumental analysis, UV-VIS spectrophotometry, liquid chromatography, electrochemistry, quality control, pharmacokinetics, bioanalysis

## Abstract

Diabetes mellitus represents a growing global health challenge, establishing oral antidiabetic drugs as a therapeutic class of paramount importance. Consequently, to ensure drug safety, therapeutic efficacy, and accurate therapeutic drug monitoring, robust, selective and highly reproducible analytical methods are indispensable. This review provides a comprehensive overview of instrumental analytical techniques developed between 1985 and 2026 for quantifying oral antidiabetic agents. Based on a literature search across major databases—including PubMed, ScienceDirect and Springer—we systematically evaluated the application of UV-VIS spectrophotometry, liquid chromatography, and advanced electroanalytical methods. The reviewed literature encompasses methodologies ranging from routine quality control of bulk powders and pharmaceutical formulations to complex bioanalytical and pharmacokinetic applications. Liquid chromatography–tandem mass spectrometry (LC-MS/MS) emerged as the gold standard for precise bioanalysis due to its superior selectivity and sensitivity in complex biological matrices. Conversely, UV-VIS spectrophotometry and electrochemical methods remain highly relevant for cost-effective routine quality control in industrial manufacturing. Methodologies are categorized by drug therapeutic class, including biguanides, sulfonylureas, DPP-4 inhibitors, SGLT2 inhibitors, meglitinides, highlighting key validation parameters such as linearity, accuracy, and limits of detection. Finally, this review outlines future directions in the field of antidiabetic drug quantification.

## 1. Introduction

The global epidemiological trajectory of diabetes mellitus is alarming, establishing it as one of the most pervasive metabolic challenges of the 21st century. Data collected through 2022 indicated that approximately 828 million adults were living with diabetes globally, reflecting an increase of about 630 million cases compared to 1990 [1]. According to the 11th edition of the IDF Diabetes Atlas published in 2025, approximately 589 million adults aged 20 to 79 were living with diabetes worldwide. While this latter figure sits lower than total population-wide models due to the strict isolation of the 20–79 age bracket, it corresponds to a global prevalence of 11.1%, meaning approximately 1 in 9 adults is affected. Long-term projections offer a concerning outlook, indicating that the total number of individuals diagnosed with diabetes will surge to approximately 853 million by 2050, affecting 1 in 8 adults [2].

Due to its complex etiology, this chronic metabolic disorder is characterized by chronic hyperglycemia resulting from defects in insulin secretion, action, or both. Clinically, the condition is categorized into major subtypes, predominantly type 1 and type 2 diabetes mellitus, alongside gestational diabetes and secondary etiologies related to exocrine pancreatic diseases. Management of these highly distinct pathophysiological profiles necessitates diverse therapeutic strategies, relying primarily on current oral antidiabetic agents to achieve glycemic control and mitigate long-term systemic complications [3,4,5].

**Figure 1:**
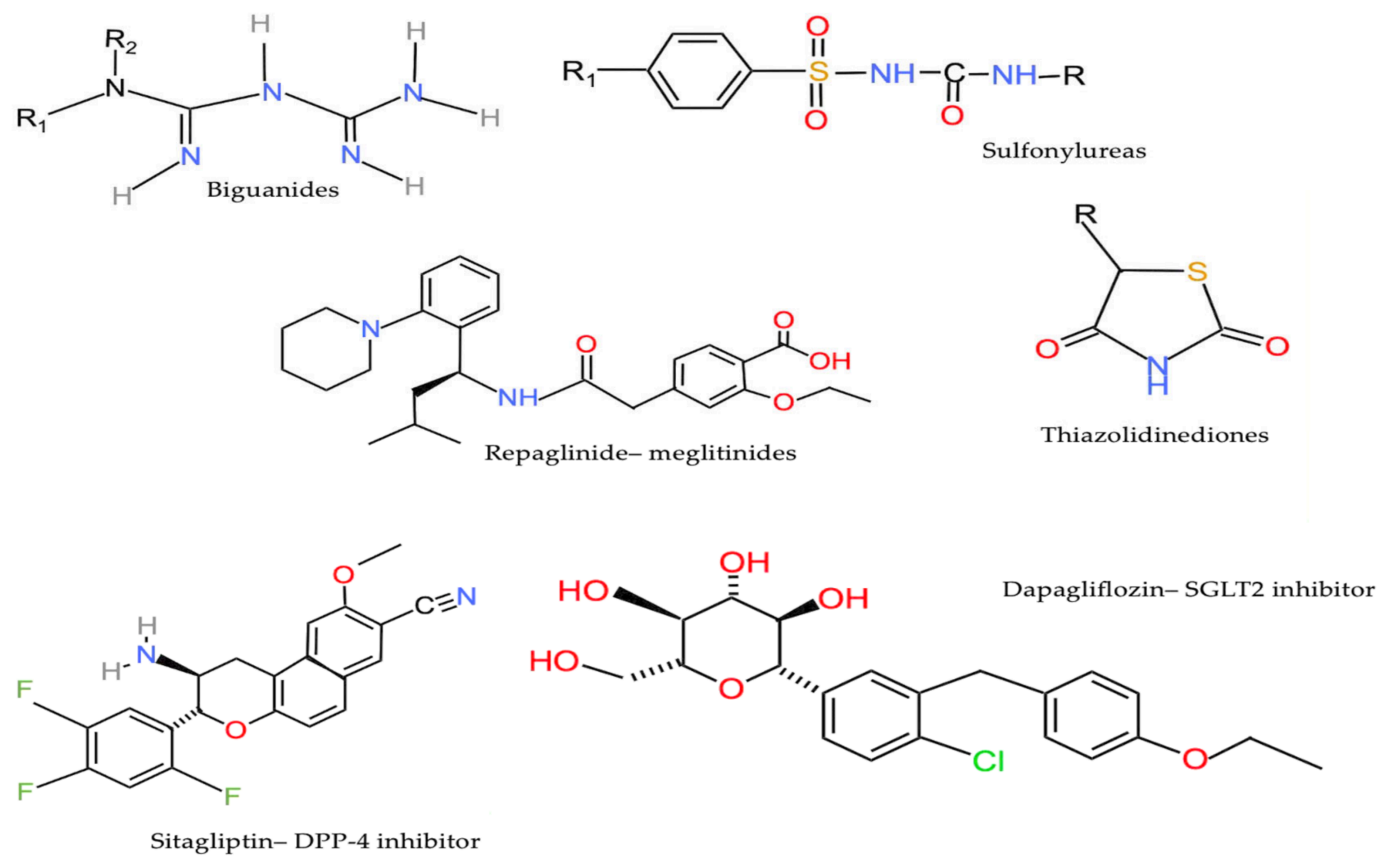
The structural formulas of the classes of oral antidiabetic agents.

In current clinical practice, the most widely used classes of oral antidiabetic drugs –as shown in Figure 1—are biguanides, sulfonylureas, glinides, thiazolidinediones, dipeptidyl peptidase-4 (DPP-4) inhibitors, and sodium-glucose cotransporter 2 (SGLT2) inhibitors. In light of the clinical limitations and scope of these oral agents, this manuscript focuses primarily on the management strategies and bioanalytical frameworks established for type 2 diabetes mellitus, in which oral therapies represent the cornerstone of metabolic regulation [6,7].

Biguanides, extensively represented by metformin alongside buformin, lower hepatic glucose secretion by inhibiting gluconeogenesis and enhancing peripheral insulin sensitivity [8]. However, the high polarity of this class poses distinct challenges in reversed-phase chromatographic separation. In contrast, sulfonylureas such as gliclazide, glimepiride, glibenclamide, and gliquidone [9] exhibit a hydrophobic nature, thereby requiring optimized separation protocols. Mechanistically related but pharmacokinetically distinct, meglitinides—primarily repaglinide and nateglinide—are characterized by a rapid onset and shorter duration of action, making them specifically suitable for targeting postprandial hyperglycemia [10]. Thiazolidinediones, which include pioglitazone, rosiglitazone, and troglitazone, rely on enhancing peripheral insulin sensitivity in adipose tissue, skeletal muscle, and the liver [11]. Structurally, specific heterocyclic cores within these molecules facilitate their monitoring via spectrophotometric and chromatographic platforms. Expanding onto current therapeutic options, sodium-glucose cotransporter 2 (SGLT2) inhibitors—clinically approved as empagliflozin, dapagliflozin, canagliflozin, and ertugliflozin—lower blood glucose levels by selectively blocking glucose and sodium reabsorption in the proximal convoluted tubules, thereby promoting glucosuria independently of pancreatic beta-cell function [12]. From an analytical perspective, their C-glucoside core and highly polar profiles lack strong chromophores, inherently necessitating advanced coupled techniques like LC-MS/MS for high-sensitivity quantification. Furthermore, the substance class of DPP-4 inhibitors, including sitagliptin, alogliptin, saxagliptin, linagliptin, and vildagliptin, enhances glucose-dependent insulin secretion, thereby minimizing the risk of hypoglycemia [13]. Structurally, these molecules feature diverse heterocyclic frameworks and functional groups that possess distinct chemical properties, allowing for their sensitive analysis through liquid chromatography.

Consequently, ensuring the rigorous quality, safety, and precise dosage of antidiabetic drugs remains an essential pharmaceutical priority. Instrumental methods—ranging from conventional UV-Vis spectrophotometry and high-performance liquid chromatography (HPLC) to advanced electroanalytical techniques—are critical for verifying active pharmaceutical ingredient (API) content. These established analytical configurations provide the precision and reproducibility required to eliminate risks of therapeutic failure or accidental toxicity in industrial manufacturing, operating in strict accordance with the validation protocols established by the International Council for Harmonisation (ICH). To address these operational standards, this review evaluates the recent literature on these quantification techniques, highlighting their contribution to both drug quality control and clinical bioanalysis, such as bioequivalence and pharmacokinetic studies. This survey of the literature encompasses in vitro dissolution, drug release profiles, and biological matrix monitoring. While UV-Vis spectrophotometry and HPLC continue to serve as baseline protocols for routine formulation testing, they set the stage for next-generation electroanalytical methods.

Concurrently, electrochemical approaches have evolved from alternative qualitative tools into standard quantitative techniques. Electroanalytical methods—such as voltammetry and potentiometry—expand conventional limits by offering low detection thresholds and high selectivity, even within complex biological or pharmaceutical matrices.

By contextualizing these emerging electrochemical platforms alongside classic spectroscopic and chromatographic techniques, this review outlines the current state of the art and establishes a foundation for future developments in accessible, cost-effective, and high-precision pharmaceutical monitoring.

## 2. Clinical Pharmacology and Bioanalytical Validation and Perspectives

### 2.1. Pharmacokinetics Aspects

From a clinical pharmacology standpoint, oral antidiabetic agents exhibit highly diverse pharmacokinetic (PK) profiles that shape bioanalytical validation protocols [14].

For instance, biguanides like metformin show negligible plasma protein binding, bypass hepatic metabolism, and are excreted unchanged in urine. This results in an apparent oral clearance that directly reflects this renal pathway. Conversely, sulfonylureas and glinides rely on extensive protein binding and hepatic clearance, though significant differences exist within these classes. For example, meglitinides cannot be collectively designated as primary substrates of cytochrome P450 (CYP)2C9; nateglinide undergoes extensive hepatic biotransformation mediated by CYP2C8 and CYP3A4. At the same time, while glipizide is rapidly metabolized by CYP2C9 into inactive metabolites, glimepiride undergoes sequential biotransformation. Gliclazide is cleared predominantly through unique hepatic oxidation pathways mediated by CYP2C19 and CYP2C9. This specific metabolic pathway requires high chromatographic selectivity to separate the parent drug from its metabolite profiles in complex biological matrices.

Absorption kinetics also vary across therapeutic classes, making uniform timeline assumptions inaccurate. Specifically, the DPP-4 inhibitor sitagliptin is rapidly absorbed, reaching peak plasma concentration (T_max_) between 1 and 4 h, whereas SGLT2 inhibitors follow different post-dose absorption rates. Under fasting conditions, SGLT2 inhibitors like canagliflozin and empagliflozin are rapidly absorbed, typically reaching peak plasma concentrations (T_max_) within 1–1.5 h. In contrast, glimepiride exhibits a slightly more prolonged and variable absorption profile, typically reaching its T_max_ between 2 and 3 h. Furthermore, gliclazide reaches T_max_ within 4 to 6 h after oral administration in the fasted state. This variability alters the required blood sampling schedules and demands sensitive analytical methods tailored to the clearance properties of each drug to accurately characterize its pharmacokinetic profile [15,16].

### 2.2. Bioanalytical Validation

Evaluating oral antidiabetic therapies requires a precise understanding of key disposition variables. Parameters such as bioavailability, peak plasma concentration (C_max_,) and time-to-peak concentration (T_max_) determine the necessary assay sensitivity and blood sampling schedules. Accounting for plasma protein binding and primary metabolic pathways is equally important to optimize sample preparation and minimize matrix interferences. Therefore, drugs with low or variable bioavailability demand highly sensitive assays to ensure accurate clinical assessment.

When transitioning from the routine quality control of bulk powders or tablet formulations to blood, serum, or plasma bioanalysis, strict adherence to regulatory guidelines is mandatory. Recent validations highlight a clear shift toward alignment with the harmonized ICH M10 guideline. These bioanalytical methods are specifically applicable to pharmacokinetic (PK), bioequivalence (BE), drug–drug interaction (DDI), and special-population studies, rather than routine therapeutic drug monitoring (TDM), which is not standard practice for oral antidiabetic regimens.

To satisfy current ICH M10 criteria, quality control and stability tests must include a minimum of three replicates per concentration level across low and high thresholds. Furthermore, bioanalytical protocols must implement strict incurred sample reanalysis (ISR) criteria, which involve reanalyzing 10% of samples for the first 1000 samples and 5% for subsequent samples. To confirm method reproducibility, at least 67% of these repeated analyses must fall within ±20% of their respective mean values.

#### 2.2.1. Matrix Effects, Carryover Challenges, and Selectivity

In bioanalysis, matrix effects (ME) represent a significant hurdle, particularly for high-throughput LC-MS/MS methods targeting biguanides and DPP-4 inhibitors. Endogenous matrix components, such as phospholipids and plasma proteins, frequently alter ionization efficiency through suppression or enhancement. To comply with ICH M10 guidelines, analytical protocols mitigate these interferences by employing advanced sample preparation like solid-phase extraction (SPE) or liquid–liquid extraction (LLE), often paired with stable isotopically labeled internal standards (SILIS).

Additionally, assay selectivity must be verified across multiple independent matrix lots to ensure the accurate separation of parent drugs from co-eluting metabolites. Instrument carryover requires careful evaluation and must remain restricted to less than 20% of the lower limit of quantification (LLOQ). This is typically achieved by optimizing organic needle wash solutions and adjusting gradient elution profiles to ensure reliable high-performance analysis [17,18].

From a clinical pharmacology perspective, validating method selectivity is vital when evaluating diabetic patients on multi-drug regimens (such as concurrent statins, antihypertensives, or antiplatelet agents). This rigorous assessment guarantees accurate drug–drug interaction (DDI) evaluations, such as identifying the metabolic inhibition of sulfonylureas by co-administered CYP2C9 inhibitors (e.g., fluconazole), which clinically triggers severe hypoglycemia [16].

#### 2.2.2. Assay Stability and Incurred Sample Reanalysis (ISR)

Evaluating the stability of antidiabetic agents under distinct storage and handling conditions is a core regulatory requirement under the ICH M10 guideline. Validation protocols must assess stock and working solution stability, short-term bench-top stability during sampling, and long-term frozen storage stability. These evaluations require a minimum of three replicates at low (LQC) and high (HQC) quality control levels across multiple freeze–thaw cycles.

For clinical evaluation, protocols incorporate incurred sample reanalysis (ISR) to verify method reproducibility using authentic clinical samples rather than spiked validation standards. Such authentic specimens frequently exhibit distinct behaviors resulting from protein binding shifts, metabolite back-conversion, or analytical interference from co-administered therapies. According to ICH M10 criteria, at least 10% of samples for the first 1000 samples, and 5% for subsequent samples, must be reanalyzed. To confirm method reproducibility and assay robustness, at least 67% of these repeated analyses must deviate by less than 20% from their original mean values [18].

#### 2.2.3. Deployment in PK/BE Studies, Therapeutic Drug Monitoring (TDM), and Special Populations

Validated bioanalytical methods are primarily deployed in pharmacokinetic (PK) and bioequivalence (BE) studies, where precise mapping of the area under the curve (AUC) and peak plasma concentration (C_max_) constitutes a regulatory prerequisite for marketing authorization. Additionally, as detailed in comprehensive reviews of contemporary bioanalytical frameworks, these high-sensitivity assays provide the structural foundation for investigating complex drug clearance networks.

In clinical research involving special populations—such as geriatric patients or individuals with renal or hepatic impairment—altered metabolic pathways significantly elevate the risk of drug accumulation and toxicity. To characterize these shifts, high-performance platforms like RP-HPLC-UV or LC-MS/MS are utilized to successfully resolve parent antidiabetic drugs from their circulating metabolites within biological matrices. Precise quantification in these controlled settings yields the critical pharmacokinetic profiles required to define safe, long-term dosing guidelines for vulnerable patient cohorts, rather than serving as a tool for routine therapeutic drug monitoring (TDM), which remains non-standard in routine antidiabetic clinical practice [19].

## 3. Clinical Treatment Outcomes of Different Antidiabetic Drugs

### 3.1. Efficacy of Different Drug Classes on HbA1c Reduction

According to available clinical datasets, all primary classes of antidiabetic medications demonstrate robust clinical efficacy in lowering glycated hemoglobin (HbA1c) levels. However, this glycemic response depends on the specific pharmacological mechanism of the class under evaluation. This therapeutic response is closely followed by sodium-glucose cotransporter 2 (SGLT2) inhibitors, which exhibit a documented reduction of 1.4%. By comparison, for intermediate agents, insulin secretagogues like sulfonylureas achieve a reduction quantified at 1.3%, while biguanides, represented by metformin, induce a 1.2% decrease.

Conversely, dipeptidyl peptidase-4 (DPP-4) inhibitors display the most modest comparative efficacy among the reviewed therapies, yielding an average reduction of 0.9% [20].

### 3.2. Cardiovascular and Renal Outcomes

Beyond standard glycemic control, large-scale cardiovascular outcome trials (CVOTs) confirmed substantial advantages regarding organ protection. For SGLT2 inhibitors, clinical evidence shows that their utilization results in a 14% reduction in major adverse cardiovascular events (MACE) and a significant 38% decrease in cardiovascular mortality in high-risk patients. Furthermore, this therapeutic class yields a 35% decline in hospitalizations for heart failure, acting through pathways independent of insulin [21].

### 3.3. Safety Outcomes, Adverse Events, and Risk Profiles

Clinical outcomes depend on the specific safety profiles, tolerability, and adverse effects associated with each agent. Clinical observations indicate that contemporary agents, specifically SGLT2 inhibitors, provide a favorable risk-to-benefit profile, successfully combining significant glycemic efficacy with proven weight-reduction advantages and a concurrently low risk of hypoglycemic events. Metformin remains the first-line choice in long-term treatment regimens, although its deployment is routinely accompanied by mild to moderate gastrointestinal side effects.

Conversely, sulfonylureas carry the highest risk-to-benefit limitations, demonstrating a severe hypoglycemia incidence rate that reaches 25%—a factor that significantly limits their utility and impacts overall patient adherence. Finally, DPP-4 inhibitors represent the most well-tolerated oral agents evaluated, demonstrating high patient compliance and a minimal side-effect profile, despite exhibiting a more modest objective efficacy regarding established glycemic endpoints [20].

## 4. Methodology

To ensure a comprehensive collection of the relevant literature, a structured search strategy was developed for this narrative review. A targeted literature search was performed across PubMed, ScienceDirect, and Springer databases and platforms covering the period from 1985 to 2026. The search strategy specifically targeted original, experimental research papers involving liquid chromatography, electrochemistry, and spectrophotometry applied to oral antidiabetic agents. Review articles, meta-analyses, and book chapters were strictly excluded from the initial phase to capture only primary analytical data. The search methodology and study selection process were established by three authors, with the subsequent data extraction performed independently by two authors and any discrepancies resolved through consensus. The detailed literature strategy and methodology are provided in Supplementary Materials.

Quality assessment and screening were performed based on predefined criteria. Studies were included if they reported validated analytical methods for oral antidiabetic agents in pharmaceutical formulations or biological matrices. In contrast, studies focusing on non-oral antidiabetic formulations and papers demonstrating poor methodological quality—specifically those lacking essential validation data, such as Limit of Detection (LOD) and Limit of Quantification (LOQ)—were strictly excluded.

Initially, a total of 4771 records were identified using the comprehensive search string: (antidiabetic OR hypoglycemic) AND (spectrophotometry OR “liquid chromatography” OR electrochemistry) AND (“quality control” OR bioanalysis OR pharmacokinetics). For the ScienceDirect database, the search was refined by limiting the subject areas to ‘’Pharmacology, Toxicology and Pharmaceutical Science’’ and ‘’Chemistry”.

Regarding the Springer search, it was refined to the disciplines of “Chemistry”, “Life Sciences”, and “Pharmacy” (specifically focusing on analytical chemistry, pharmacology, and pharmacy subdisciplines). Across all platforms, the publication language was limited to English. After removing 2326 duplicate records manually and excluding 1745 records during a preliminary review (comprising other review articles and book chapters), 700 reports were screened based on title and abstract, leading to the exclusion of 500 irrelevant records. The remaining 200 reports were assessed for full-text eligibility. A total of 61 reports were excluded for specific reasons (incomplete validation data, non-oral formulations, or lack of bioanalytical parameters such as LOD and LOQ). Finally, 139 studies met all inclusion criteria and were selected for final incorporation in this review.

## 5. Instrumental Techniques for Quantification of Oral Antidiabetic Agents

### 5.1. Spectrophotometric Methods

Regarding the spectrophotometric determination of oral antidiabetic drugs, polar solvents such as ethanol, methanol or distilled water are commonly used. A critical aspect in solvent selection is ensuring minimal absorbance within the ultraviolet-visible (UV-Vis) spectral range. Consequently, this optical transparency minimizes background interference during the quantification of the target compounds.

Methanol is one of the most widely employed solvents in UV-Vis spectrophotometry due to its negligible absorbance across the primary measurement wavelengths. The primary objective of these analytical evaluations is to quantify the active pharmaceutical ingredient (API), either as a monotherapy or co-formulated with other oral antidiabetic drugs, within common matrices encompassing powdered tablet formulations and complex biological specimens.

Oral antidiabetic agents possess diverse physicochemical properties that facilitate characterization via classical spectrophotometric techniques. The structure of these pharmaceutical substances is also characterized by chromophoric functional groups capable of inducing electronic transitions in the UV-Vis range [22,23,24,25,26].

This analytical versatility is well-demonstrated across various structural classes quantified via methanol-based UV-Vis spectrophotometry. For instance, pioglitazone, a thiazolidinedione derivative, exhibits distinct spectral properties that permit direct, conventional, spectrophotometric quantification without requiring analytical signal enhancement through chemical derivatization [27].

Similar approaches have proven effective for less frequently studied agents, such as the first-generation sulfonylurea chlorpropamide. While chromatographic methods are generally recognized for higher resolution and selectivity, the consistent validity, linearity, and accuracy achieved across these diverse drug classes underscore UV-Vis as a robust, cost-effective alternative [28,29]. Beyond methanol, other simple polar solvents are equally effective for modern therapeutic classes; for instance, the quantitative determination of dapagliflozin utilizes ethanol or distilled water as the solvent, under conventional spectrophotometric conditions [30,31]. With a maximum absorption wavelength (λ_max_) at 234 nm, this method offers excellent sensitivity, specificity, precision, and accuracy, without suffering from interference from common table excipients [30].

Owing to its well-established operating principles, spectrophotometry remains a highly precise, reproducible, and accessible instrumental technique for pharmaceutical analysis. This applicability extends to comparative quality control, particularly in assessing bioequivalence and formulation consistency across different manufacturers [32].

**Figure 2:**
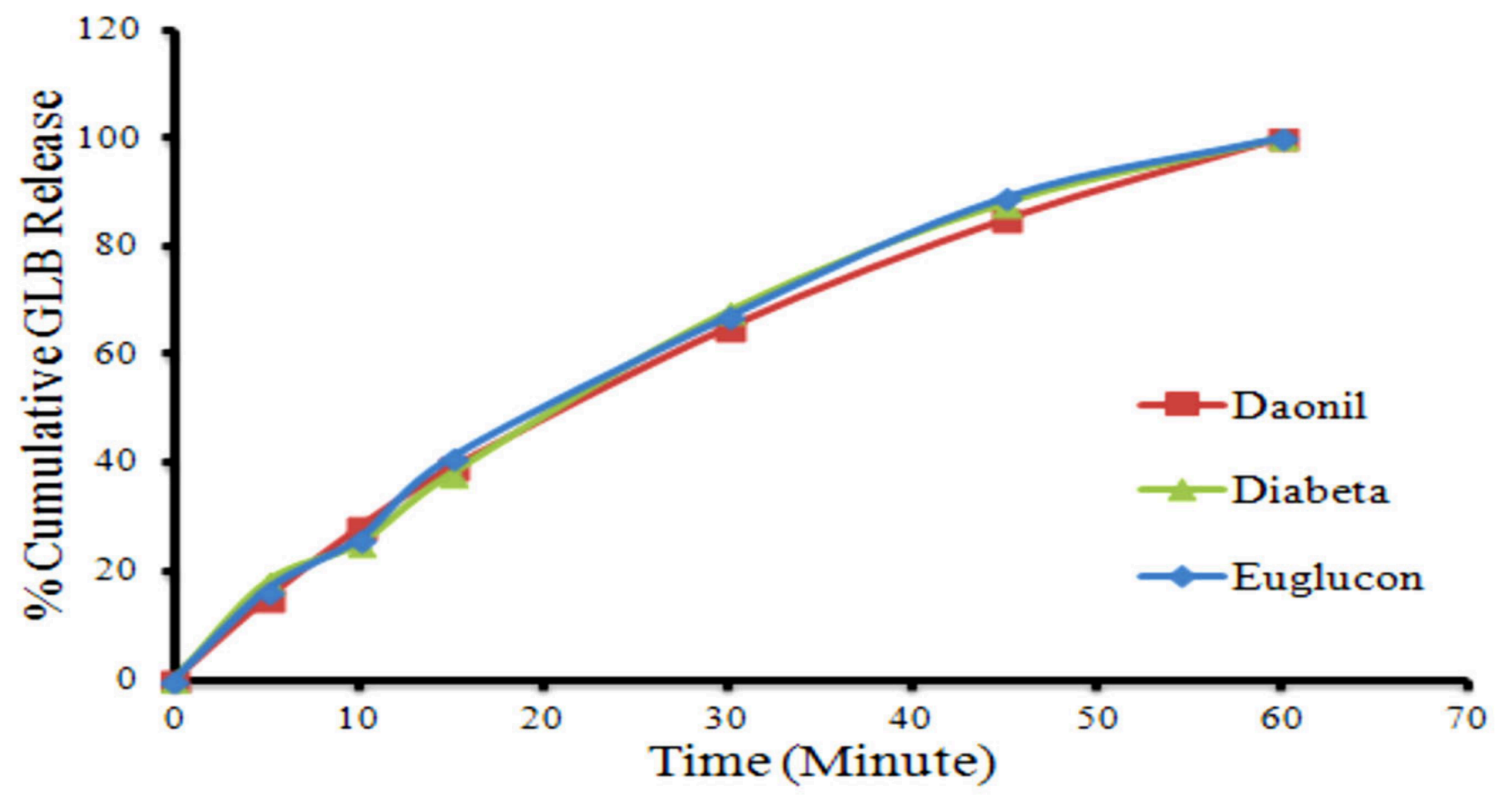
In vitro dissolution profiles of glibenclamide from three different commercial brands. Adapted from an open access source [32].

As illustrated in Figure 2, the in vitro dissolution profiles of glibenclamide across the three commercial brands (Daonil, Diabeta, and Euglucon) exhibit nearly superimposable release kinetics over the 60 min evaluation period. Each formulation displays a rapid initial release phase—exceeding over 40% cumulative dissolution within the first 20 min–followed by a sustained, parallel progression toward maximum dissolution at approximately 100%.

This overlapping kinetic behavior underscores a high level of uniformity in manufacturing quality, excipient composition, and active pharmaceutical ingredient (API) particle size distribution across the different manufacturers. To put these findings into a broader critical perspective, this high formulation consistency aligns with published international databases; for instance, comparative spectrophotometric dissolution screenings of alternative generic glibenclamide brands have similarly confirmed robust bioequivalence and aligned release kinetics relative to the innovator product [33]. While minor regional variances in dissolution efficiency can sometimes arise due to localized manufacturing standards or specific excipient choices [34], the uniform patterns demonstrated here strongly validate the reliability of conventional UV-Vis methods for industrial quality control.

From a biopharmaceutical perspective, such comparable dissolution rates strongly suggest that these generic products are expected to exhibit equivalent in vivo performance and therapeutic bioequivalence, ensuring predictable drug absorption and minimizing the risk of fluctuating blood glucose control when switching between brands. Moreover, the structural and physicochemical diversity of antidiabetic agents has driven the development of derivative spectrophotometry as an advanced resolution tool. These methodologies facilitate the precise quantification of oral antidiabetic drugs regardless of their therapeutic substance classes. For instance, these techniques enable the simultaneous analysis of multiple active substances used in diabetes management, even in the presence of degradation products generated during sample preparation or long-term storage of the pharmaceutical dosage form [35].

This analytical approach is equally applicable to the evaluation of sulfonylureas within complex pharmaceutical formulations containing various excipients. Although these manufacturing additives can potentially interfere with spectrophotometric signals, thereby compromising analytical accuracy, targeted assay optimization can effectively mitigate these matrix effects. Consequently, achieving high selectivity requires the precise adjustment of critical analytical parameters, including solvent composition, molar ratio, and pH thresholds [36].

From the perspective of green chemistry and environmental safety, derivative spectrophotometry offers distinct advantages by minimizing organic solvent consumption and utilizing non-toxic reagents [37].

Expanding beyond the quantification of single-component formulations, UV-Vis spectrophotometry has proven highly effective for the simultaneous determination of multi-component systems, particularly within co-formulations of glycemic regulators and antioxidant compounds. A prominent clinical case involves the simultaneous quantification of the sulfonylurea gliclazide and the flavonoid quercetin. By leveraging the spectral differentiation of the two analytes, this approach completely bypasses the need for preliminary chromatographic separation steps, thereby streamlining the analytical workflow while maintaining high analytical accuracy [38]. To overcome the selectivity constraints inherent to conventional spectrophotometry when analyzing multi-component systems, recent studies have increasingly turned toward chemometric tools. These mathematical and statistical frameworks resolve overlapping spectra, enabling the simultaneous quantification of two or more antidiabetic agents. The primary advantage of these determinations lies in providing high-throughput analysis, thereby maintaining robust analytical performance [39,40].

Extending this principle from the simultaneous quantitative analysis of two oral antidiabetic drugs, chemometrics can also be applied to quantify a mixture of an oral antidiabetic agent and the calcium channel blocker, verapamil. Even though they do not belong to the same therapeutic class and exhibit entirely different mechanisms of action, they can be present in a binary mixture, making their simultaneous determination feasible. This study highlights the versatility and flexibility of analytical chemistry in quantitative analysis [41].

Specifically, Bhaskar et al. [40] and Hassaninejad-Darzi et al. [39] applied chemometric platforms to enhance spectrophotometric resolution, demonstrating significant advantages over conventional zero-order analysis. In contrast to computationally heavy multivariate calibrations, mathematical derivative spectra offer a simpler, cost-effective alternative for selective signal enhancement.

Beyond chemometric methods and the advantages these techniques provide in the simultaneous quantification of pharmaceutical agents, the literature also reports the Q-analysis method, which is based on measuring absorbance values at two wavelengths corresponding to the analyzed compounds. One of these wavelengths corresponds to the isoabsorptive point, and using this spectral information enables the determination of the concentration of each compound [42]. Concurrently, the method of simultaneous equations is often compared with Q-analysis, as both rely on absorbance measurements at key strategic wavelengths to circumvent physical separation [43,44,45,46]. For instance, Reichal et al. [42] successfully preserved the simplicity of UV-VIS spectrophotometry by applying the Q-analysis method at the isoabsorptive point, significantly decreasing the data processing time. While this approach successfully isolates key analytical steps, its reliance on fixed wavelengths demands precise calibration to prevent spectral drift.

When the absorption spectra of the analytes overlap, it can become difficult to accurately distinguish between the spectral data of two oral antidiabetics. To address this, the literature also describes an innovative method using discrete and continuous wavelet transforms. These are mathematical techniques applied to process the spectrophotometric signal. Accordingly, the developed approach had appropriate analytical validation and is very time-efficient, as the literature reveals an increasing trend toward the use of mathematical principles to improve the efficiency of multicomponent analysis. Moreover, given that the interpretation of spectral data demands advanced mathematical techniques, it is considered highly difficult to apply these methods in routine analytical laboratories [47].

To address specific formulations, previous studies used two comparative methods to simultaneously and selectively quantify linagliptin and metformin, thereby successfully overcoming the limitations caused by spectral overlap. By developing this method, the authors demonstrated satisfactory analytical performance without chromatographic separation [48]. Even though most studies focus on the simultaneous determination of two oral antidiabetic agents, recent investigations have extended this scope to quantify three compounds simultaneously. Nevertheless, the clinical utility of such methods is often constrained, as the presence of numerous interfering substances within tablet formulations often limits the selectivity of analytical techniques [49]. Furthermore, advanced mathematical techniques applied to the simultaneous quantification of oral antidiabetics exhibit varying error margins; thus, method selection depends strictly on the required selectivity, precision, and error susceptibility [50]. Alternatively, the area under the curve (AUC) method is widely utilized for the quantitative analysis of binary mixtures, such as glibenclamide and alogliptin. This approach relies on measuring the individual absorbance of each active substance over a specific wavelength range, followed by AUC computation. The key advantages of this technique include high accuracy, rapidity, and reduced analysis time, while eliminating the need for prior chemical separation. This places AUC alongside other powerful chemometric tools, such as derivative spectrophotometry and wavelet-transform-based methods [46].

Within the field of spectrophotometry, mixed hydrotropy has emerged as an innovative approach to enhance analyte solubility in aqueous media through hydrotropic agents, such as urea and sodium acetate. Consequently, the reliance on expensive and toxic organic solvents is significantly reduced [51]. In this context, the recent literature emphasizes the selection of spectrophotometric quantification techniques that favor eco-friendly, non-toxic alternatives—a shift aligned with modern green analytical principles.

While instrumental analysis dominates, titrimetry remains a valuable tool for quantifying oral antidiabetic agents via specific bromometric pathways. Furthermore, novel methodologies continue to evolve, leveraging chemical reactions designed to enhance analytical signal intensity, as summarized in Table 1.

Characterized by their pronounced coloration, ion-pair complexes can enhance the analytical signal in the quantification of pharmaceutical substances such as oral antidiabetic drugs. This analytical approach offers a rapid and cost-effective alternative to chromatographic methods. However, a notable limitation regarding analysis time arises because additional steps are involved prior to spectrophotometric determination, such as complex formation, extraction using organic solvents and the optimization of critical reaction conditions (including pH, solvent type, and time).

**Figure 3:**
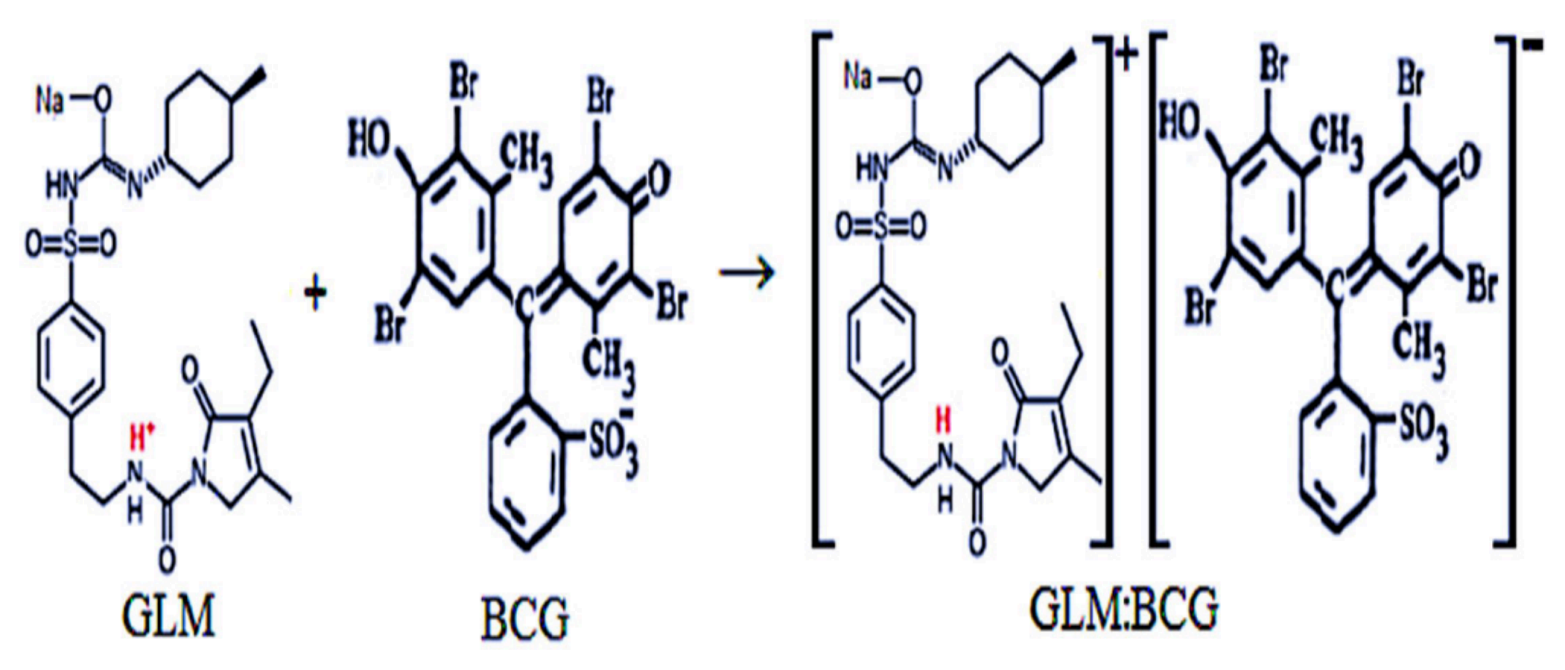
Chemical reaction involved in the formation of the glimepiride–bromocresol green complex. Adapted from an open access source [57].

In addition to optimizing the experimental conditions for the glimepiride-bromocresol green ion-pair complex, as illustrated in Figure 3, current research frequently extends this methodology to other oral antidiabetic agents, or explores alternative mechanisms, such as the oxidative coupling reactions employed for linagliptin. These strategies successfully lower the limits of detection (LOD) and quantification (LOQ) while improving precision. When compared to techniques that involve advanced mathematical applications—which may be less suitable for routine analyses in pharmaceutical laboratories—chemical approaches like metal complexation or dye binding consistently yield stable compounds and a distinct amplification of the analytical signal.

Green chemistry principles have reshaped modern pharmaceutical analysis, offering the possibility of performing spectrophotometric determinations using less toxic reagents or minimizing the use of toxic organic solvents, while maintaining reliable analytical performance and improving sensitivity [59]. Moreover, the recent literature highlights the transition toward eco-friendly reagents for the quantitative analysis of multi-component formulations, such as combinations of dapagliflozin and omeprazole in synthetic mixtures [60]. Such approaches successfully demonstrate that cost reduction and environmental safety can be achieved without compromising chromatographic or spectrophotometric efficiency.

To expand the scope of green spectrophotometry, attention has also shifted toward the meglitinide therapeutic subclass. In particular, a novel approach utilizes an interaction with a colored compound, substituting conventional dyes with a reagent system consisting of chloranilic acid and dichloro-dicyano-benzoquinone. This interaction leads to the formation of a charge-transfer complex where the generated stable ionic radical species are detectable in the visible range, thereby providing satisfactory analytical performance [61].

Beyond routine quality control, the determination of oral antidiabetic drugs has been extended to biological matrices, such as human plasma or urine. These applications underscore the versatility of spectrophotometric detection when coupled with selective extraction protocols, allowing for the reliable tracking of pharmaceutical agents even in complex biological environments [62].

To improve selectivity and specificity, UV-VIS spectrophotometry frequently incorporates advanced derivatization strategies. Unlike conventional protocols relying on a single complexation reaction (either with a transition metal or with an organic dye, such as eosin), current methodologies utilize ternary systems. In these approaches, multiple simultaneous interactions lead to the formation of antidiabetic agent–metal–dye complexes. Thus, enhanced selectivity, specificity, and accuracy are demonstrated through the amplification of the analytical signal resulting from this complex formation [63].

Metformin, a foundational therapeutic agent within the oral antidiabetic class, belongs to the biguanide therapeutic subclass, and is widely used in current treatment regimens due to its effectiveness in regulating blood glucose levels. Consequently, it has been the subject of extensive quantitative and qualitative analyses, reflecting its relevance in pharmaceutical sciences. Owing to its distinct physicochemical properties, it can be quantified using UV-VIS spectrophotometry as a single active substance in the composition of certain tablets or in combination with other oral antidiabetics and agents from other therapeutic subclasses [64,65]. Consequently, a critical evaluation of the specific analytical frameworks applied to metformin underscores their diverse methodological parameters and performance outcomes, as can be seen in Table 2.

As a compelling alternative, a recent approach applied to a mixture containing canagliflozin and metformin incorporates a preliminary sample enrichment step. Unlike Raveendra et al. [66], who augmented analytical selectivity via mathematical algorithms, Wafaa et al. [70] focused primarily on direct sample modification. Specifically, the extract obtained containing canagliflozin was processed to selectively enrich the SGLT-2 inhibitor while reducing interference from metformin. This methodological path bypasses complex mathematical derivatives, or cost-intensive instrumentation, demonstrating that reliable determination remains achievable using standard laboratory infrastructure while maintaining satisfactory linearity, precision and accuracy. Consequently, it highlights a clear divergence in current analytical strategies, where some methods harness multivariate mathematical tools to refine spectral data, whereas others modify the sample chemically to enhance selectivity.

The trend toward increasing complexity in spectral interpretation persists with the implementation of multivariate techniques. While certain protocols aim to improve selectivity via derivative spectrophotometry, complementary frameworks enhance accuracy by simultaneously exploiting multiple spectral features. However, the execution of such protocols depends heavily upon advanced chemometric software, thereby restricting their applicability in routine analytical practice. Understanding these parameters is imperative, as they directly dictate the choice of extraction solvents and the optimization of chromogenic reactions in spectrophotometric protocols.

Despite the inherent analytical limitations of spectrophotometry, the recent literature indicates its viability for quantitative determination in complex matrices. For instance, Joshi et al. [71] shifted focus from pharmaceutical formulations toward physiological environments, quantifying saxagliptin in simulated gastric juice. This method demonstrated good linearity, precision, and accuracy under simulated physiological conditions. The significance of this approach lies in its ability to evaluate drug behavior under bio-relevant settings rather than focusing solely on quality control, achieving this via cost-effective and accessible instrumentation.

Spectrophotometric methods can also evaluate the behavior of drugs, such as repaglinide, by assessing their stability and detecting potential degradation pathways. These investigations were conducted under stress conditions, including variations in pH, temperature, or exposure to oxidizing agents. By monitoring the active pharmaceutical ingredient (API), the authors aimed to leverage UV-VIS spectrophotometry for the comprehensive assessment of structural integrity, thereby expanding its applications [72,73].

Shifting the analytical focus from quantification to structural integrity assessment underscores the versatile utility of this methodology under diverse stress testing frameworks. The same approach is also applicable to monitoring the dissolution profiles of pioglitazone capsules, providing pharmaceutically relevant information regarding the release profile of the active substance (whether conventional, sustained, or modified release formulations). Accordingly, the reported method measured absorbance at a specific wavelength of 270 nm to determine the cumulative amount of drug dissolved in the medium. Spectrophotometrically, this study encompasses both drug quantification and physicochemical characterization, which are essential for formulation design [74]. In this case, a more specific analytical focus has been directed toward monitoring biopharmaceutical behavior through the evaluation of the release profile of oral antidiabetic agents, thereby distinguishing this study from prior research centered solely on the characterization of specific pharmaceutical formulations.

From an alternative perspective, UV-VIS spectrophotometry can be utilized either to quantify drug substances in pharmaceutical formulations or biological matrices, or to evaluate the stability and release profiles of various oral antidiabetic formulations. Indeed, Vaishnav et al. [75] shifted the traditional focus of spectrophotometry—conventionally restricted to quality control—toward assessing the therapeutic efficacy of repaglinide in type 2 diabetes management. Given the satisfactory results in terms of precision, linearity, and accuracy, the method was validated for clinical pharmacological applications. By bringing this approach closer to the clinical field, their findings underscore the broader implications of instrumental analysis in therapy evaluation. While some characterizations remain anchored strictly in the realm of pharmaceutical technology, shifting this focus toward clinical monitoring underscores the broader practical implications of instrumental analysis in therapy evaluation.

Applied spectrophotometry encompasses a variety of methods, ranging from conventional UV-Vis spectrophotometry to advanced techniques, depending on the analytical conditions, raw materials, and reagents utilized. The primary advantages of these analytical approaches include low operational cost, robust experimental reproducibility, and the availability of various reagents to optimize analytical performance.

Furthermore, chromatographic methods are indispensable when superior precision, sensitivity, and selectivity are required. Consequently, the literature emphasizes several comparisons between the quantification of oral antidiabetic drugs in solid dosage forms such as tablets, using both UV-VIS spectrophotometry and High-Performance-Liquid Chromatography (HPLC) [76]. For instance, comparative evaluations between UV-VIS spectrophotometry and HPLC have been reported for analyzing rosiglitazone (a thiazolidinedione) and metformin hydrochloride. These studies highlight the various advantages and limitations of each instrumental technique. Concurrently, UV-VIS spectrophotometry is characterized by simplicity and rapidity, specifically suitable for routine pharmaceutical analysis. The concordant quantitative results underscore the strong complementarity of both analytical methods [77].

### 5.2. Column Chromatography Methods

As previously outlined, oral antidiabetic agents represent a diverse pharmacological class comprising substances with distinct physicochemical and pharmacological properties. Thus, for both the quantitative and qualitative determination of these compounds, the continuous development and selection of sensitive, selective, and versatile chromatographic methods remain essential, especially given the inherent structural diversity among the analytes described above.

The current literature demonstrates that liquid chromatography– specifically high-performance liquid chromatography (HPLC), ultra-high-performance liquid chromatography (UHPLC), and liquid chromatography–tandem mass spectrometry (LC-MS/MS)– represents the predominant analytical platform for the quantitative analysis of low-molecular-weight antidiabetic agents, a trend largely driven by significantly reduced analysis time and method versatility [78].

From an analytical perspective, these chromatographic methods are critical for isolating active pharmaceutical ingredients from complex matrices containing excipients, formulation degradation products, and co-administered drugs. Consequently, these methodologies play a dual role by assessing the long-term stability of pharmaceutical products and enabling the precise quantification of these target compounds in complex biological matrices, such as human plasma, to accurately characterize their pharmacokinetic properties [79].

The global prevalence of type 2 diabetes drives an increasing demand for rapid and sensitive protocols suitable for routine quantitative workflows. Consequently, modern research focuses on aligning chromatographic methods with green analytical chemistry principles. Therefore, minimizing or replacing organic solvents with eco-friendly alternatives is a priority, alongside shortening analysis times [78].

Concurrently, Lam et al. [79] highlighted the expansion of liquid chromatography into bioanalysis. Their work demonstrates the capacity of chromatographic methods to effectively isolate oral antidiabetic agents from excipients, co-administered pharmaceutical substances, and potentially harmful degradation products. Unlike other authors who focused on general protocols for quantifying these substances, they highlighted the versatility of chromatographic platforms and the practical relevance of these approaches.

For the quantitative determination of SGLT-2 inhibitors, chromatographic separation typically relies on reversed-phase high-performance liquid chromatography (RP-HPLC) coupled with ultraviolet (UV) detection. Thus, standard protocols utilize nonpolar C18 stationary phases under isocratic elution modes. The developed methods consistently yield analytical results in accordance with regulatory frameworks, demonstrating satisfactory linearity and reproducibility which are essential parameters for routine quality control of pharmaceutical products [80,81,82,83,84]. Compared to alternative methodologies characterized by complex configurations, these studies point toward an approach focused on simplicity, linearity, and, importantly, reproducibility, in accordance with ICH guidelines. A practical application of these validated methods involves the quality control comparison of two marketed brands, both labeled to contain 10 mg of dapagliflozin, using HPLC-UV [85].

Moreover, the pharmaceutical substances from the therapeutic subclass of sodium-glucose co-transporter 2 (SGLT-2) inhibitors can also be detected using tandem mass spectrometry coupled with electrospray ionization. By comparing the results from different detection techniques, the study demonstrates that ionization-based detection offers enhanced selectivity and specificity due to the confirmation of the structural identity of the analyzed compounds, even though the associated HPLC separation conditions were similar [86]. In this regard, such approaches bridge the gap between routine pharmaceutical quality control and advanced structural elucidation, significantly expanding the analytical scope of modern column chromatography.

**Figure 4:**
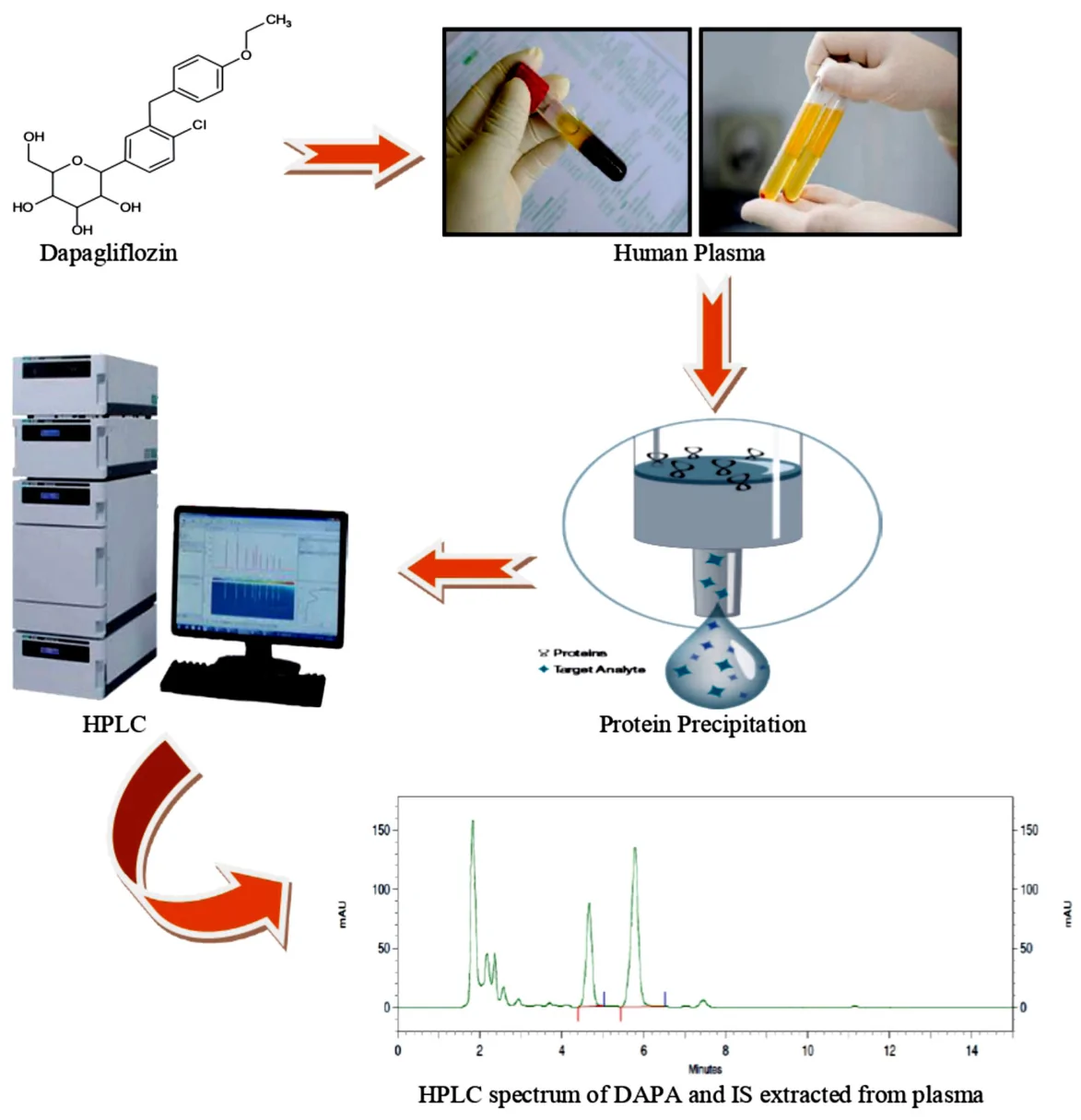
Schematic representation of the steps involved in the quantification of dapagliflozin in human plasma. Reprinted from an open access source [87].

Beyond traditional quantitative analyses, SGLT-2 inhibitors like dapagliflozin are increasingly monitored within bioanalytical frameworks, especially in human plasma (Figure 4). Shifting to clinical matrices necessitates rigorous optimization to meet stringent selectivity, sensitivity, and lower limits of quantification. Consequently, the primary advancement involves adapting standard reversed-phase protocols to accommodate highly complex biological backgrounds. Achieving this requires a meticulous selection of organic modifiers and specialized stationary phases to minimize matrix suppression effects while maximizing signal-to-noise ratios and overall resolution [87].

At the same time, LC-MS/MS methodologies can also be extended to the quantification of oral antidiabetic agents in human plasma, providing a much more selective approach. Beyond optimizing the chromatographic conditions required to ensure satisfactory separation, tandem mass spectrometry serves as an advanced detection platform, enhancing method selectivity and sensitivity [88,89]. While standard reversed-phase high-performance liquid chromatography (RP-HPLC) protocols offer robust quantification for routine assays, recent frameworks strongly advocate for this hybrid configuration. This paradigm shift underscores the analytical advantages of mass spectrometry in managing complex biological backgrounds. Consequently, these comparative evaluations clearly demonstrate the structural elucidation capabilities afforded by adopting ionization-based detection in modern bioanalysis.

Other published studies highlight the importance of chromatographic methods in evaluating the pharmacokinetic profiles of these substances in animal models, particularly rodents. In this regard, an LC-MS/MS method was proposed for the quantitative analysis of ipragliflozin in rat plasma. This technique is characterized by selectivity, sensitivity, and extended detection capability compared with alternative methods limited to the quantification of drug substances in pharmaceutical formulations. Operationally, the authors utilized liquid–liquid extraction (LLE) to isolate the analyte from complex endogenous components. This step was followed by chromatographic separation on a nonpolar C18 column and positive electrospray ionization detection, placing the protocol among modern bioanalytical methodologies for SGLT-2 inhibitors [90].

While maintaining a focus on the applications of instrumental analysis to evaluate the pharmacological properties of oral antidiabetic drugs, a recently developed bioanalytical method investigated drug-food interactions, specifically between empagliflozin and grapefruit juice, known for its enzymatic inhibitory effect on cytochrome P450 isoenzymes. Thus, in addition to its ability to quantify SGLT-2 inhibitors, this chromatographic technique can also be used to evaluate the drug’s pharmacokinetic profile [91].

These targeted investigations emphasize the critical role of advanced chromatography in mapping complex pharmacokinetic profiles, spanning from absolute bioavailability to formulation-dependent drug-food interactions [90,91]. Specifically, optimizing these instrumental configurations allows for a more comprehensive assessment of the intricate in vivo behavior and metabolic clearance of SGLT-2 inhibitors under real-world dietary conditions.

Furthermore, liquid chromatography can also be used to evaluate the chemical stability of dapagliflozin. In this regard, recent research utilizes high-performance liquid chromatography coupled with a diode array detector and mass spectrometry (HPLC-DAD-MS) methods, which aim to separate the antidiabetic drug from its degradation products and subsequently identify these compounds. This strategy yields satisfactory performance in terms of selectivity, specificity, and detectability, providing insights into both the degradation products of dapagliflozin and its stability behavior under various stress conditions [92].

Another approach regarding the stability study of dapagliflozin involves a UPLC-PDA method, utilizing a phenyl column and an isocratic mobile phase of acetonitrile:water (70:30, *v*/*v*) with detection at 230 nm. Thus, both the drug substance and three of its impurities were simultaneously determined, with the method demonstrating high sensitivity and being validated in accordance with ICH guidelines. In this context, evaluating the correlation between chromatographic analysis and the in vitro assessment of impurity toxicity remains highly relevant. This is because one of the impurities was responsible for producing significant cytotoxic effects in 3T3 mouse fibroblast cells. Therefore, the investigation focused not only on demonstrating the analytical performance of the UPLC method but also on evaluating the safety profile of the drug [93].

Chromatographic analysis aimed at identifying the degradation products of empagliflozin was performed using UHPLC coupled with a quadrupole time-of-flight tandem mass spectrometry (QTOF/MS) detector. Focusing specifically on the structural characterization of degradation products rather than toxicological effects, this study investigated empagliflozin as the target analyte [94]. While structural identification of degradation products remains a primary objective [92,94], achieving satisfactory analytical sensitivity and separation represents an equally critical benchmark in stability studies.

Regarding alternative SGLT2 inhibitors, the degradation products of empagliflozin were evaluated utilizing an HPLC method to assess stability, highlighting the potential of chromatographic techniques to characterize the behavior of SGLT-2 inhibitors under stress conditions. This aspect distinguishes this study from routine assay protocols applied to pharmaceutical formulations [95].

**Figure 5:**
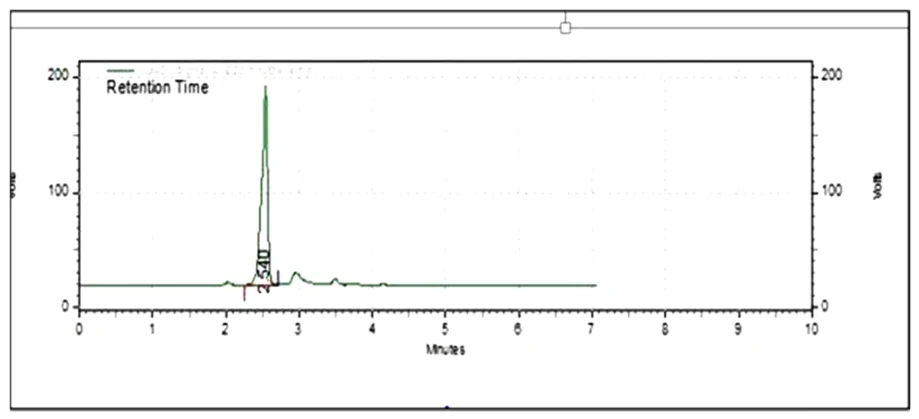
Representative HPLC chromatogram of empagliflozin. Adapted from an open access source [97].

The focus shifts from the routine quantification of dapagliflozin to the quantitative analysis of its enantiomer, as well as associated impurities, using pure dapagliflozin as the raw material. Therefore, compared to the other articles discussed, which focus on column chromatography, the relevance of this study lies particularly in highlighting the stereochemical properties of the active substance and in assessing the impurities of this drug analyte. Moreover, this sample analysis by RP-HPLC represents an environmentally sustainable protocol, utilizing less toxic mobile phases than those used in conventional chromatographic methods [96]. This critical approach underscores a dual analytical objective: the simultaneous resolution of the dapagliflozin enantiomer and its associated impurities. Such applications reinforce the importance of optimizing chromatographic parameters to provide high chemoselectivity and trace-level detectability, as demonstrated in Figure 5.

**Figure 6:**
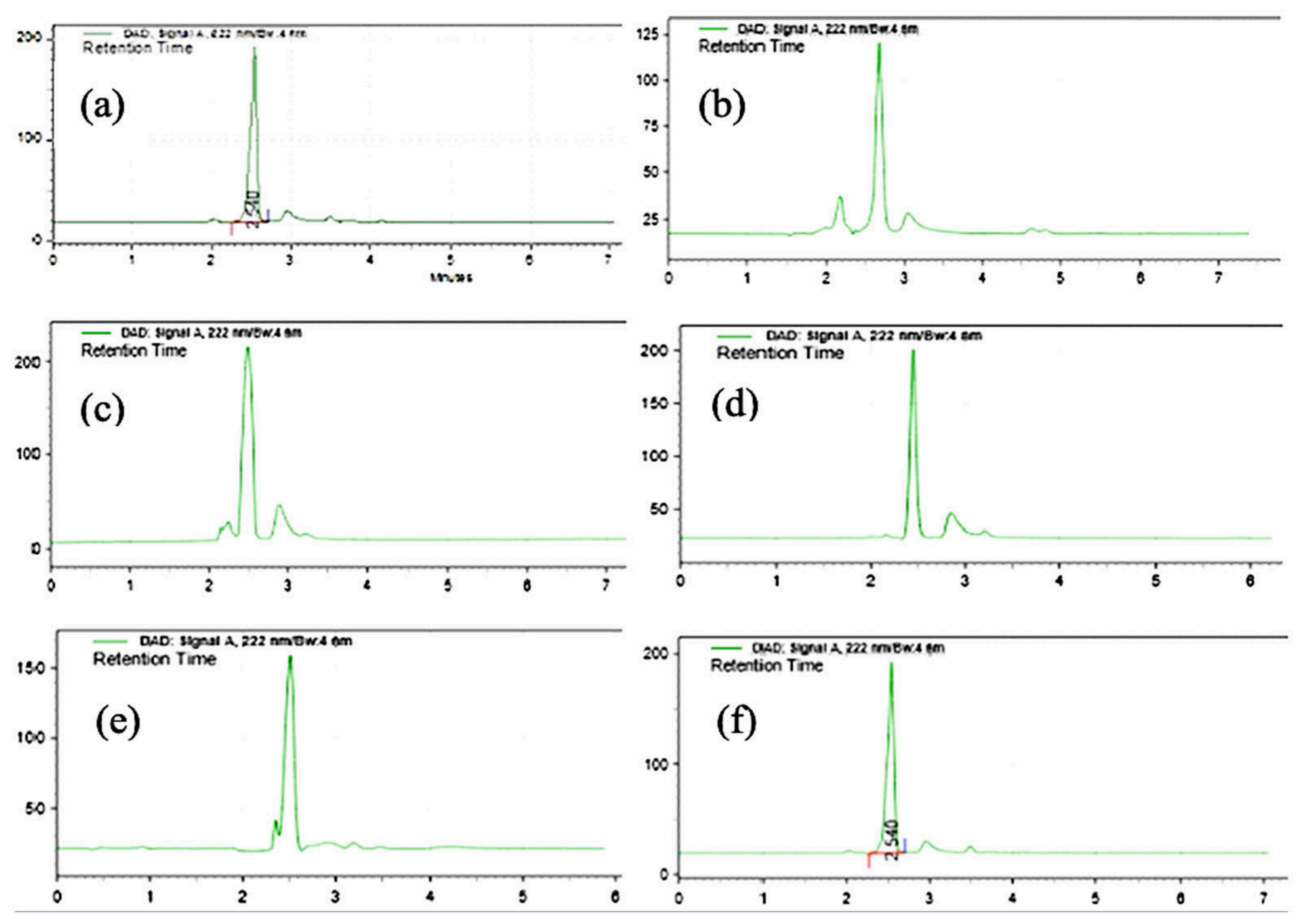
Representative HPLC chromatograms of empagliflozin demonstrating chemical stability under diverse stress testing conditions: control (**a**), acidic (**b**), basic (**c**), oxidative (**d**), photolytic (**e**), and thermal (**f**) environments. The primary peaks correspond to the intact active pharmaceutical ingredient, while secondary peaks signify resolved degradation products. Adapted from an open-access source [97].

The chromatographic profiles presented in Figure 6 provide critical insights into the stability-indicating capability of the reviewed liquid chromatography protocols. Under baseline control conditions, the drug exhibits a singular, well-resolved peak verifying its chemical purity. Upon exposure to forced degradation conditions, a demonstrable decrease in the parent drug’s peak intensity occurs concurrently with the baseline resolution of newly emerged degradation products at distinct retention times. This performance underscores the high selectivity of the method, confirming its suitability for routine pharmaceutical quality control and stability monitoring.

Quantitative determination of antidiabetic drugs can be performed in mixtures consisting of two or more compounds. For instance, an RP-HPLC method for the simultaneous determination of metformin and dapagliflozin was systematically developed based on the Quality by Design (QbD) concept. The approach highlights not only the simultaneous analysis of two drug substances, but especially the fact that the method has been optimized to be more robust and more controllable under various laboratory conditions. Consequently, the QbD paradigm proves highly effective for both multi-analyte systems and the rigorous standardization of single pharmaceutical substances [98,99].

The chromatographic methods developed for separating various oral antidiabetic drug mixture are detailed in Table 3.

The analytical methods used for the quantification of thiazolidinediones and related compounds across various samples are detailed in Table 4.

Abdelgawad et al. [114] expanded this analytical framework by developing a cost-effective and eco-friendly chromatographic protocol for monitoring the pharmacokinetic behavior of pioglitazone. Through the minimization of hazardous organic modifiers, this developed approach successfully addresses the classic complexities associated with laborious quantitative assays.

In this context, navigating the balance between chromatographic platforms requires a critical understanding of their trade-offs based on the specific analytical goal such as routine quality control, bioanalysis, or stability-indicating assays. For clinical bioanalysis within complex matrices like human or rat plasma, LC-MS/MS setups deliver the ultimate sensitivity and rapid run times often under 12 min, which are crucial for pharmacokinetic studies. However, they carry a specific sample preparation to ensure adequate matrix-effect resistance, alongside high equipment costs. Conversely, RP-HPLC-UV offers lower operational expenses and permits straightforward protein precipitation protocols. When the analytical scope shifts toward stability-indicating assays and impurity profiling, where separating closely eluting degradation products is mandatory, advanced multi-dimensional platforms like 2D-UHPLC-HRMS provide superior selectivity and high matrix-effect resistance, fully justifying their extended run times and elevated costs. Finally, for the routine quality control of standard tablet formulations where matrix interferences are negligible, conventional isocratic RP-HPLC-UV provides the optimal equilibrium, reducing the sample-preparation burden to basic dissolution and filtration at an accessible cost.

Extending this analytical scope beyond the standard quantitative analysis of the active pharmaceutical ingredient, the literature suggests the possibility of profiling target metabolites—specifically *N*-desmethylrosiglitazone and *p*-hydroxyrosiglitazone—present in human plasma using LC-MS/MS. To achieve this, a comprehensive method was developed to evaluate both the parent drug and its metabolites simultaneously [115]. While alternative investigations primarily focused on characterizing degradation products under stress conditions, this mass spectrometric approach provided a significantly more comprehensive pharmacokinetic evaluation. By enabling the simultaneous quantification of the parent compound alongside the profiling of its active metabolites, the protocol maximized operational efficiency through minimal human plasma volumes and simple sample preparation via deproteinization.

This integration of high-efficiency separation and advanced detection is mirrored in the quantification of antidiabetic agents belonging to the therapeutic substance class of DPP-4 inhibitors. Whether utilizing RP-HPLC or UPLC, these frameworks share uniform analytical considerations, such as combining a nonpolar C18 column with a polar or semi-polar mobile phase, and subsequent detection in the ultraviolet range or by mass spectrometry, depending on the intended purpose [116,117,118,119]. This methodological synergy aligns with the majority of investigations aiming both to analyze oral antidiabetic drugs in various pharmaceutical formulations and to quantify or identify them in biological fluids [120,121].

Advanced chromatographic methods have been developed to analyze critical impurities, such as degradation products or enantiomeric isomers of semaglutide. Within this highly challenging analytical context, separating and characteristically profiling dextrorotatory (D) and levorotatory (L) isomers is essential, given their identical molecular masses and highly subtle structural variances. To overcome these limitations, high-resolution techniques utilize RP-UPLC separation coupled with HRMS for accurate mass measurement and structural confirmation. Following the isolation of specific isomeric fractions, hydrolysis with deuterated hydrochloric acid combined with chiral derivatization and RP-UPLC-MS/MS analysis enables the precise identification of D-Ser8, D-His1, and D-Asp9 impurities in semaglutide formulations [122]. Consequently, driven by the critical need to fully map the safety profile of these complex pharmaceutical substances, a clear trend toward leveraging ultra-high-performance chromatography for the selective separation and stereochemical evaluation of antidiabetic isomers has emerged as a state-of-the-art approach in the recent literature.

Expanding beyond the pharmacological field, which coexists with instrumental analysis, pharmaceutical technology applies methods to investigate the behavior of linagliptin—namely, the drug’s release profile from tablets—in a dissolution test. To this end, the analytical performance of this dissolution assay was rigorously monitored on the linagliptin tablets using HPLC-UV. Consequently, this chromatographic method can be used as a complementary tool in the quality control of linagliptin tablets, thereby highlighting the role of instrumental analytical methods in evaluating biopharmaceutical properties [123].

While conventional evaluations primarily focus on elucidating the pharmacokinetic properties of oral antidiabetics, contemporary approaches increasingly utilize advanced chromatographic analysis to complement the biopharmaceutical profiling of various solid dosage forms. Given that linagliptin exists in enantiomeric form, various methodologies for the enantioselective separation of these isomers have been systematically explored. For instance, by utilizing a coated amylose chiral stationary phase, this chromatographic protocol offers a highly effective methodology designed not only to quantify the active pharmaceutical ingredient but also to stringently monitor enantiomeric purity by separating linagliptin from its undesired optical isomer [124].

Applying a similar chiral separation principle, repaglinide was subsequently analyzed using an enantioselective approach. However, this method distinguishes itself by correlating the chromatographic analysis with the biological activity of the stereoisomers of the target antidiabetic agent using molecular docking studies on carbonic anhydrase II. Furthermore, the separation was performed on a less commonly used Chiralcel^®^ OD-RH chiral column, with n-hexane:2-propanol 95:5 (*v*/*v*) as the mobile phase and detection at 240 nm [125]. Specifically, this molecular docking analysis revealed the 3D binding pose within the active site of the target protein and mapped the 2D interaction profile with key amino acid residues.

In this context, evaluating the stereochemical behavior and achieving the precise structural identification of isomers remains paramount, as these spatial configurations directly dictate pharmacological efficacy and biological activity.

Consequently, while oral antidiabetic agents belonging to the sulfonylurea therapeutic subclass have been less frequently studied over time using spectrophotometric or electrochemical methods, the literature reports a substantial transition toward robust chromatographic frameworks, such as HPLC, RP-HPLC, LC-MS/MS, or UPLC. Due to their polar nature, these specific blood-glucose-lowering agents are efficiently quantified utilizing standard C18 or C8 stationary phases coupled with polar or semi-polar mobile phases, frequently operated under isocratic elution modes. While routine quality control workflows typically employ various commercial tablets or bulk powders [126,127], these advanced chromatographic methods can be simultaneously applied to evaluate biopharmaceutical properties. More specifically, the focus is directed on a tablet’s capacity to disintegrate in the digestive tract and be released for absorption throughout the body [128].

Regarding biological matrices, sample preparation protocols often involve deproteinization, followed by reversed-phase separation under comparable conditions and subsequent detection via UV or mass spectrometry [129,130]. Strategic harmonization of these chromatographic parameters ultimately enables the simultaneous quantification of antidiabetic agents across distinct therapeutic subclasses, thereby significantly expanding the pharmacokinetic evaluation of these multi-drug regimens [130,131,132,133]. Beyond pharmacokinetic profiling, chromatographic methods are also suitable for assessing the stability of sulfonylureas or for identifying impurities with toxicological implications. Such evaluations can be performed either as part of routine quality control or following forced degradation of tablet formulations to accurately assess degradation behavior.

Under similar column chromatographic conditions, these developed methods offer a rapid and environmentally friendly alternative while simultaneously evaluating the stability of pharmaceutical formulations [134,135]. Furthermore, these bioanalytical determinations can be performed by processing human plasma or urine using a C18 or C8 column integrated into a chromatographic system coupled with a diode array detector [136].

Based on a comprehensive literature assessment, while chromatographic workflows provide definitive separation profiles for sulfonylureas, broadening their monitoring scope necessitates the integration of alternative, non-destructive analytical strategies.

### 5.3. Electrochemical Methods

Due to the increasing clinical demands of oral antidiabetic drug therapy, alternative analytical configurations are continuously explored to enhance sensitivity and throughput. In this context, electrochemical methodologies offer a highly effective alternative by exploiting the redox properties of target pharmaceutical substances at the electrode-solution interface. Consequently, these electroanalytical designs are easily adapted for quality control testing of pharmaceutical formulations, as well as high-throughput screenings in complex biological or environmental matrices.

Among these, the most widely used analytical techniques are cyclic voltammetry, square-wave voltammetry, differential pulse voltammetry, stripping voltammetry, and amperometry. Regarding the principles of these methods, the literature indicates that cyclic voltammetry is predominantly used to investigate the redox behavior of the substance of interest, while the other techniques offer superior sensitivity and specificity, thereby achieving lower detection limits [137].

Within this analytical framework, metformin holds a special place in the pharmacological literature, making this therapeutic agent an important analytical focus. In addition to the methods described and analyzed earlier, quantification by electrochemical means provides a major advantage because, besides pharmaceutical products and biological fluids, environmental samples can also be investigated. This indicates a significant contribution of electrochemistry to detecting this pharmaceutical substance as a contaminant in environmental samples [138].

Among the innovations introduced in the literature in this analytical domain, one method worth mentioning is based on the electrocatalytic effect of Cu(II) on a glassy carbon electrode modified with a Cu-BTC (HKUST-1) metal–organic framework. Utilizing this structured electrochemical sensor architecture, the developed electroanalytical method exhibits optimal specificity, high reproducibility and long-term stability [139].

Analogously, the focus shifts from the glassy carbon electrode to a carbon paste electrode, serving as a conductive support, whose surface is modified with a multicomponent system consisting of bacterial nanocellulose, copper oxides, and silver nanoparticles. Through this strategy, the anodic signal of metformin is enhanced by the newly synthesized electrode, resulting in a more sensitive and specific analytical response [140]. In addition to these configurations, analyzing drug substances in pharmaceutical products can be successfully achieved using a carbon paste electrode modified with a hybrid system based on a phosphorus-doped material, namely P-g-C3N4, and a Cu(II)-based metal–organic framework (MOF).

**Figure 7:**
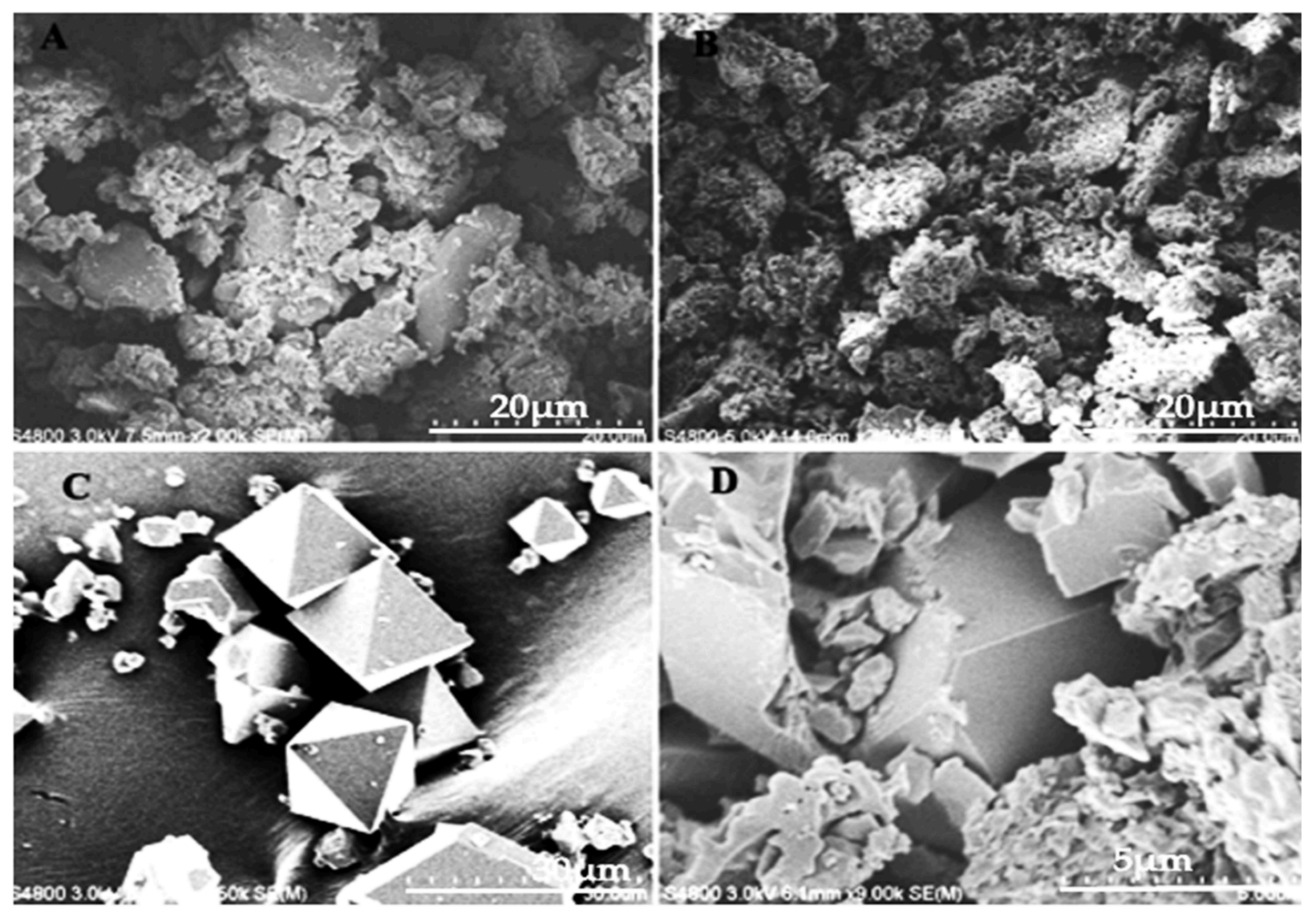
SEM images illustrating the morphology of the nanocomposite material used in the development of an electrochemical sensor for metformin. Adapted from an open access source [141].

The copper-containing composite promotes the electrochemical oxidation of metformin, whereby the chemically modified electrode yields a 39-fold increase in anodic peak current compared to the unmodified sensor. Consequently, strategies relying on the chemical modification of the supporting electrode demonstrate a vastly superior analytical performance over conventional protocols employing bare electrodes [141], as illustrated in the SEM images in Figure 7.

Alternative electroanalytical approaches for the quantitative determination of metformin incorporate the deployment of a carbon cloth electrode modified with a molecularly imprinted polymer, nickel cobaltite, and graphene oxide nanorods. Within this design, the literature highlights a novel approach that improves selectivity and amplifies the electrochemical signal through the synergistic effect of graphene oxide and nickel cobaltite nanorods within the molecularly imprinted polymer matrix. This composite architecture significantly enhances the anodic peak current, leading to lower quantification limits of oral antidiabetic agents [142].

For bioanalytical validation, metformin quantification can be performed using human urine and metformin tablets as sample matrices. The method employed a boron-doped diamond electrode to ensure reliable detection of this drug. By subsequently modifying this electrode with Nafion, a synthetic ionic polymer, interference from the highly complex biological matrix was reduced. In contrast to alternative sensor configurations, this electroanalytical assembly demonstrates excellent operational simplicity by using an optimized polymer film casting that eliminates time-consuming surface pretreatment steps [143].

Electrochemistry is characterized by its versatility and the advanced use of materials in the quantitative analysis of oral antidiabetic agents, particularly metformin. The glassy carbon electrode was modified with a multicomponent system consisting of carbon black, hydrated ruthenium dioxide, and the synthetic ionic polymer, Nafion. The analytical method used was adsorptive stripping voltammetry, which is based on the adsorption of the analyte onto the electrode surface, followed by voltammetric determination. The primary advancement relies on incorporating hydrated ruthenium dioxide, which grants extended electrocatalytic stability and maintains sensor sensitivity for at least three weeks. Consequently, this robust surface circumvents the need for frequent electrode fabrication, ensuring an economical workflow backed by highly favorable validation metrics [144].

Current research trends in this field focus extensively on increasing the analytical signal intensity and improving the selectivity by deploying electrodes modified with nanomaterials, metal oxides, polymers, or hybrid composites. Each modifier offers a distinct structural design, critical for quantifying analytes like metformin even in complex biological matrices. Concurrently, this increasingly pronounced shift from conventional to modified electrodes demonstrates that electrochemical platforms are no longer perceived merely as niche alternatives, but as a dominant, high-performance direction for the quantification of target analytes.

An alternative approach for the quantitative determination of metformin involved the employment of a pencil graphite electrode, along with the use of an alkaline medium in the presence of 0.1 M NaOH. This protocol offers significant originality and simplicity, as it does not involve a modified electrode, but rather an electrochemically activated one. Thus, this biguanide assay format emphasizes operational efficiency and raw-material accessibility, effectively reducing analysis times while eliminating hazardous chemical regeneration steps. Furthermore, electrochemical characterization maps the clear oxidation mechanism of metformin, documented at an anodic potential of 1.28 V [145].

**Figure 8:**
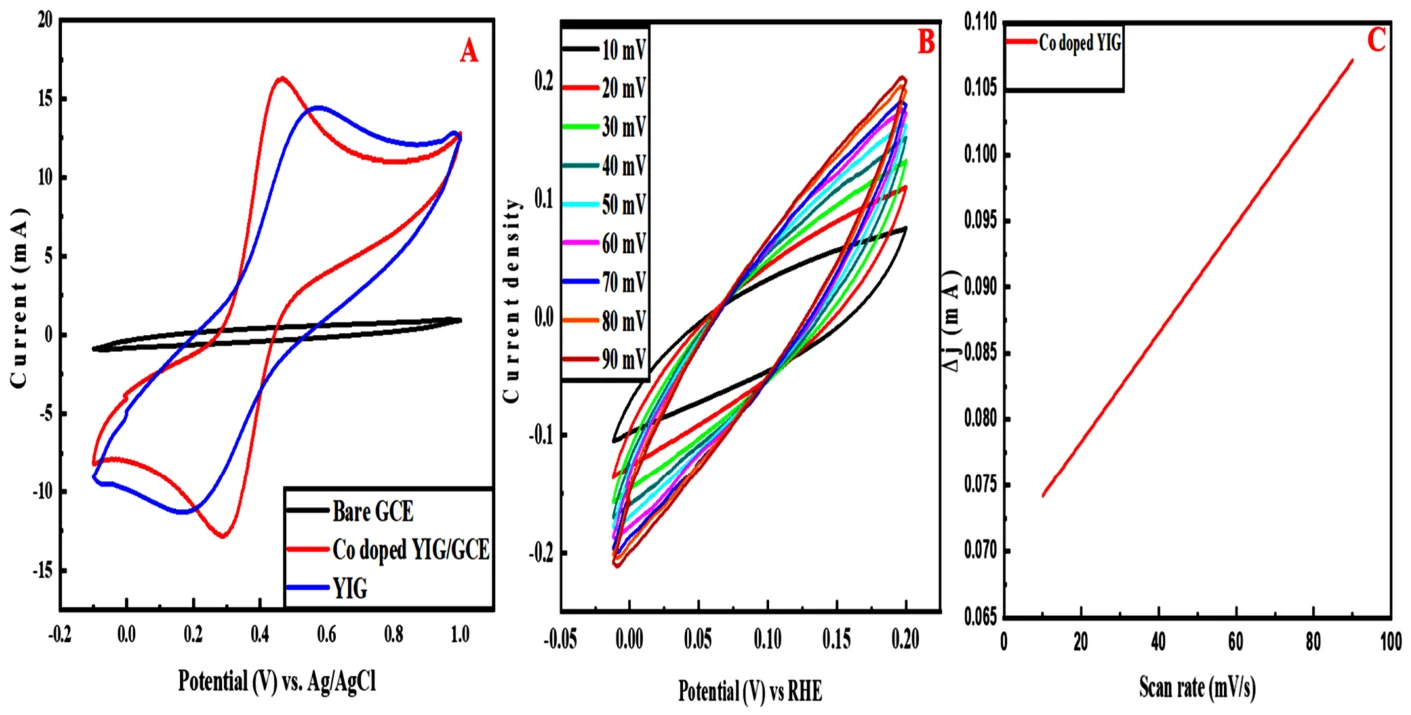
Preliminary sensor characterization steps (prior to metformin analysis) in 0.1M potassium ferricyanide with 0.1M KCl: (**A**) CV (Cyclic voltammetry) profiles showing conductivity variations between bare, YIG, and Co–doped YIG/GCE systems to demonstrate the enhanced electrochemical activity achieved after modification; (**B**) ECSA (electroactive surface area) evaluation using cyclic voltammetry at different scan rates from 10 to 90 mV/s; (**C**) linear calibration curve derived for ECSA calculations. Such foundational optimization steps are critical for achieving high–sensitivity metformin screening. Adapted from an open access source [146].

To further expand the diversity of sensor modification strategies, a glassy carbon sensor was modified with a garnet material, obtained by incorporating iron and yttrium, doped with cobalt. This integration provides the electrode with more pronounced electroactive properties, thereby ensuring enhanced analytical sensitivity [146], as illustrated in Figure 8.

A significant focus in current research is dedicated to developing modified architectures that are more sensitive and capable of detecting small amounts of analyte. In addition to experimental methods, a promising approach consists of modifying electrochemical electrodes using cobalt(III) oxyhydroxide, yielding electrochemically reactive species that function optimally in neutral and slightly alkaline environments. From a theoretical standpoint, although the electrochemical signal generated by the sensor is expected to be clear and easy to interpret, this modeling emphasizes that the possibility of system instability must also be considered [147].

Regarding baseline comparisons, classical designs using unmodified substrates offer straightforward operation with satisfactory analytical performance. Conversely, alternative strategies rely on specialized surface-modifying nanomaterials to significantly amplify the electrocatalytic response. While these advanced modifications optimize sensitivity, they inherently increase fabrication costs compared to standard, non-modified graphite electrodes. Shifting attention to the sulfonylureas, their electroanalytical determination frequently employs carbon paste, glassy carbon electrodes, or associated nanoparticles [137]. Although historical protocols utilized mercury film electrodes for these assays, modern developments strongly phase them out due to environmental toxicity, favoring non-hazardous polymer-based modifiers that align with sustainable analytical principles.

Focusing on gliclazide, the selectivity and specificity of its determination continue through the modification of electrodes, such as glassy carbon electrodes, with molecularly imprinted polymer films, including polypyrrole. This approach offers the opportunity to attribute two major roles to the polymer matrix immobilized on the electrode: enhanced selectivity compared with other methods that do not use modified sensors, and specificity through the phenomenon of molecular recognition, typical of molecularly imprinted polymers [148].

Modified electrochemical sensors are gaining ground, in most cases, instead of conventional unmodified ones due to their superior properties that enhance the analytical response. In this regard, gliclazide and glibenclamide were quantitatively determined using a carbon paste sensor modified with ZnIn_2_S_4_ nanoparticles and an ionic liquid, namely 1-butyl-3-methylimidazolium hexafluorophosphate. The developed method showed satisfactory validation parameters. The functional components involved in this surface modification synergistically lower charge-transfer resistance, making the platform highly suitable for the electroanalytical trace detection of these two oral sulfonylureas; based on the experimental data, this configuration exhibits reproducible and highly sensitive detection capabilities [149].

In the literature, these systems are routinely preferred to optimize analytical parameters compared with those of unmodified substrates, as is also noted in the case of gliclazide, which could be quantified with lower detection thresholds using an electrode modified with a multicomponent mixture of magnetite (Fe_3_O_4_) and graphene oxide [150]. Specifically, this electrochemical platform is fabricated as a modified carbon paste electrode by mixing Fe_3_O_4_@GO nanostructures with graphite powder and paraffin oil, allowing for the sensitive voltametric trace detection of the analyte using automated instrumentation.

In the case of sulfonylureas, the focus is not exclusively on the detection of analytes, but also on enhancing the selectivity of the methods by introducing sensors modified with nanoparticles, ionic liquids, or molecularly imprinted polymer films. Thus, more specific recognition of the analyte can be achieved, an aspect that is particularly important when analyzing oral antidiabetic agents in mixtures with other pharmaceutical substances from the same therapeutic class or from different therapeutic subclasses.

While modified electrodes are widely recognized for their superior sensitivity, satisfactory analytical performance for key antidiabetic agents—such as glimepiride, glibenclamide, pioglitazone, and rosiglitazone— can still be achieved using conventional setups. Specifically, selecting standard glassy carbon or carbon paste electrodes coupled with cyclic voltammetry or differential pulse voltammetry offers a highly cost-effective and practical alternative for routine frameworks [151].

To evaluate alternative thiazolidinediones, pioglitazone was also analyzed electrochemically using cyclic voltammetry, differential pulse voltammetry, linear voltammetry, and square-wave voltammetry. These methods aimed to investigate the electrochemical behavior as a function of pH, the nature of the buffer system used, concentration, and scan rate. Based on the collected data, the authors sought to highlight the oxidation mechanism of this thiazolidinedione, as its quantification does not specifically require modified electrodes—which can be quite costly—but rather relies on the inherent electrochemical properties of the antidiabetic agent [152].

Complementary studies have likewise established matrix-independent protocols for quantifying oral antidiabetic drugs without requiring surface functionalization, achieving highly accessible and reproducible analytical data. Additionally, introducing a conventional, non-modified sensing substrate alongside straightforward electrochemical activation offers an alternative to complex setups. By coupling established techniques with simple sensors, these investigations demonstrate that reliable analytical performance can be successfully achieved through routine, economic, and streamlined workflows.

In contrast to thiazolidinediones, DPP-4 inhibitors receive less attention in the electrochemical literature regarding methods for their quantitative determination. In the case of sitagliptin, one effective analytical method involved the use of molecularly imprinted polymer nanoparticles with electrochemical activity immobilized on a screen-printed platinum electrode [153].

**Figure 9:**
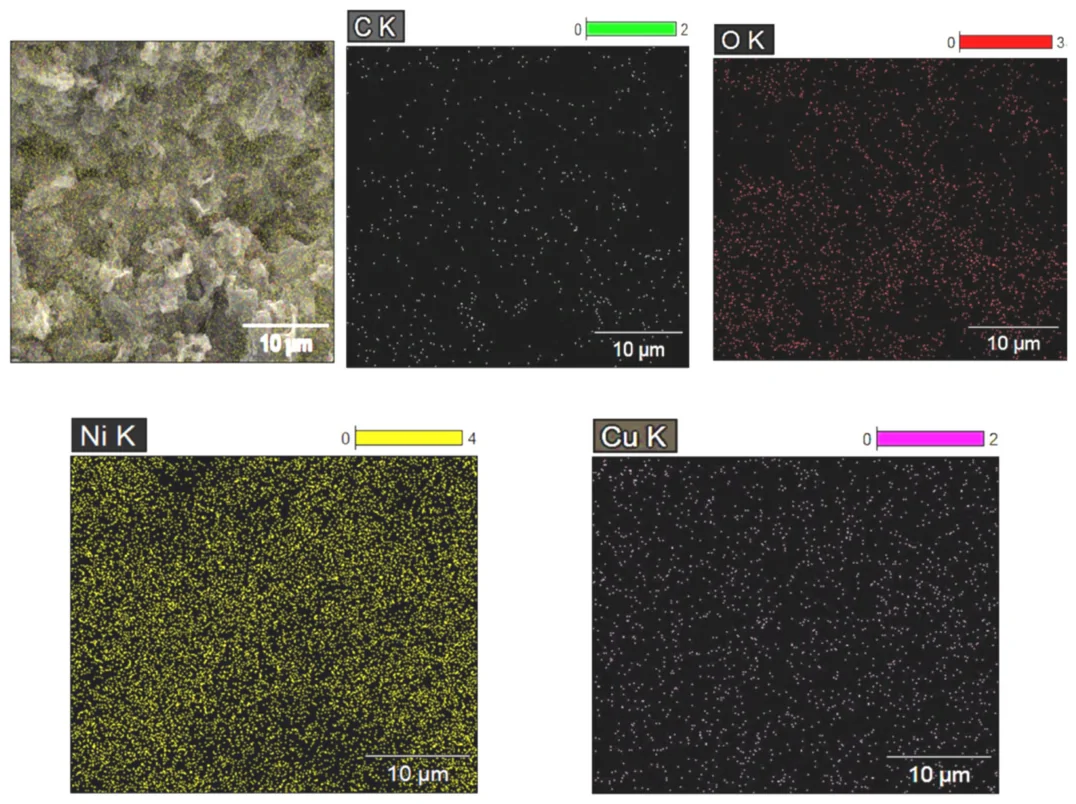
EDX elemental mapping of the GO/NiO/CuO nanocomposite used as an electrode-modifying material for electrochemical detection of an antidiabetic drug (where carbon is shown in green, oxygen in red, nickel in yellow, and copper in pink). Adapted from an open access source [154].

Although the literature most often mentions the modification of the sensors with two combined compounds, electrochemical analyses can be performed using a three-component modification. Specifically, surface modification of glassy carbon substrates utilizing a ternary composite of graphene oxide, nickel oxide, and copper oxide significantly amplifies the electrocatalytic response paths. This composite sensor targets critical performance optimization in sitagliptin sensing while maintaining highly accessible and stable operational conditions [154]. The nanocomposite elemental mapping is illustrated in Figure 9.

Electroanalytical research increasingly emphasizes the use of nanomaterial-modified electrodes for enhanced linagliptin detection. For instance, a glassy carbon electrode modified with 1T-MoS_2_ nanoparticles and L-cysteine demonstrated a significant increase in the electroactive surface area, thereby facilitating electron transfer at the sensor interface [155].

Beyond standard quantification, the electrochemical behavior and oxidative properties of linagliptin were investigated using a carbon paste electrode (CPE) modified with iron(III) oxide nanoparticles. This surface configuration amplified the electrochemical response at the electrode interface, yielding a stronger analytical signal via square-wave voltammetry.

From a clinical standpoint, a simultaneous determination in co-formulations remains essential. To address this requirement, protocols propose cost-effective and sensitive voltammetric alternatives capable of quantifying linagliptin in the presence of metformin and glucose without prior chromatographic separation [156].

**Figure 10:**
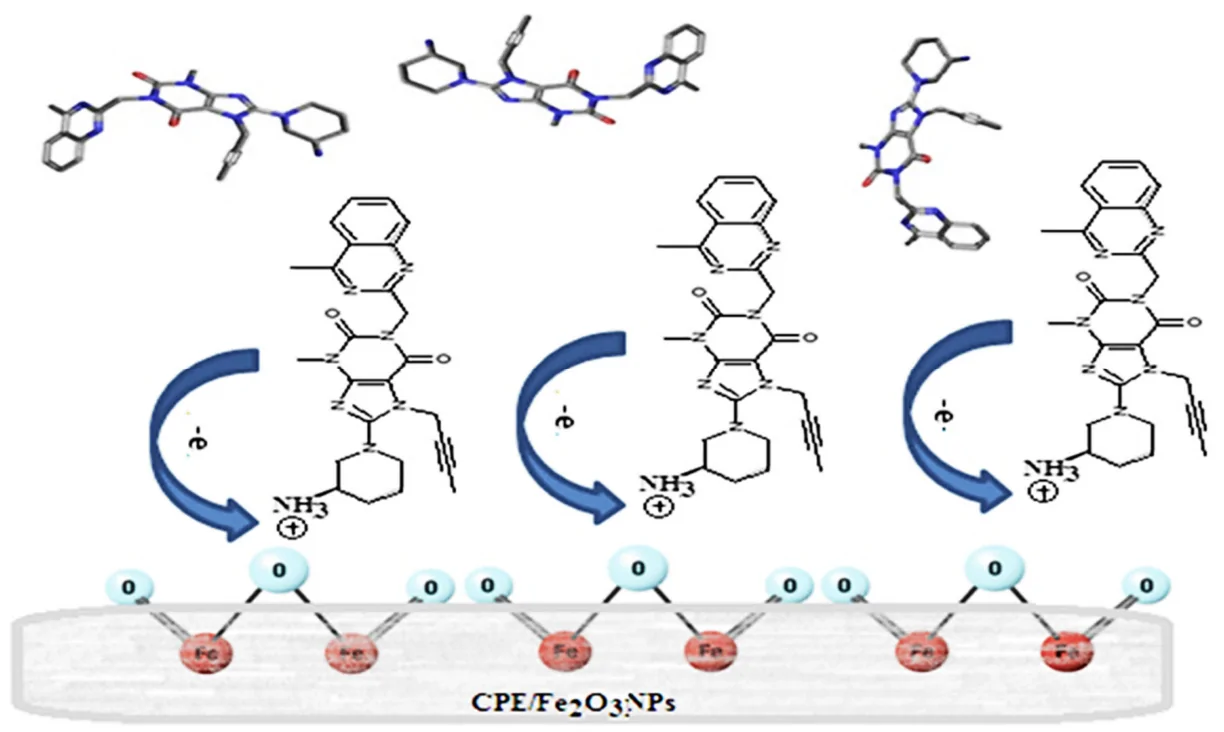
The mechanism of reaction between linagliptin and the CPE/Fe_2_O_3_NPs electrochemical sensor. Reprinted from an open access source [156].

As shown in Figure 10, linagliptin molecules interact with the electrode surface, resulting in an electron transfer that generates an electrical current—an analytical signal used to quantify the antidiabetic agent.

Moreover, the simultaneous determination of linagliptin and dapagliflozin in human plasma was achieved using a pencil graphite electrode modified with a multicomponent system composed of hematoxylin, chitosan, and zirconium nanoparticles. While hematoxylin and chitosan enhance the overall electroactivity, the zirconium nanoparticles expand the active contact area between the target analytes and the electrode surface. This functionalization significantly improves electroselectivity, enabling the quantification of multiple drug substances with an analytical performance comparable to classical chromatographic methods [157].

**Figure 11:**
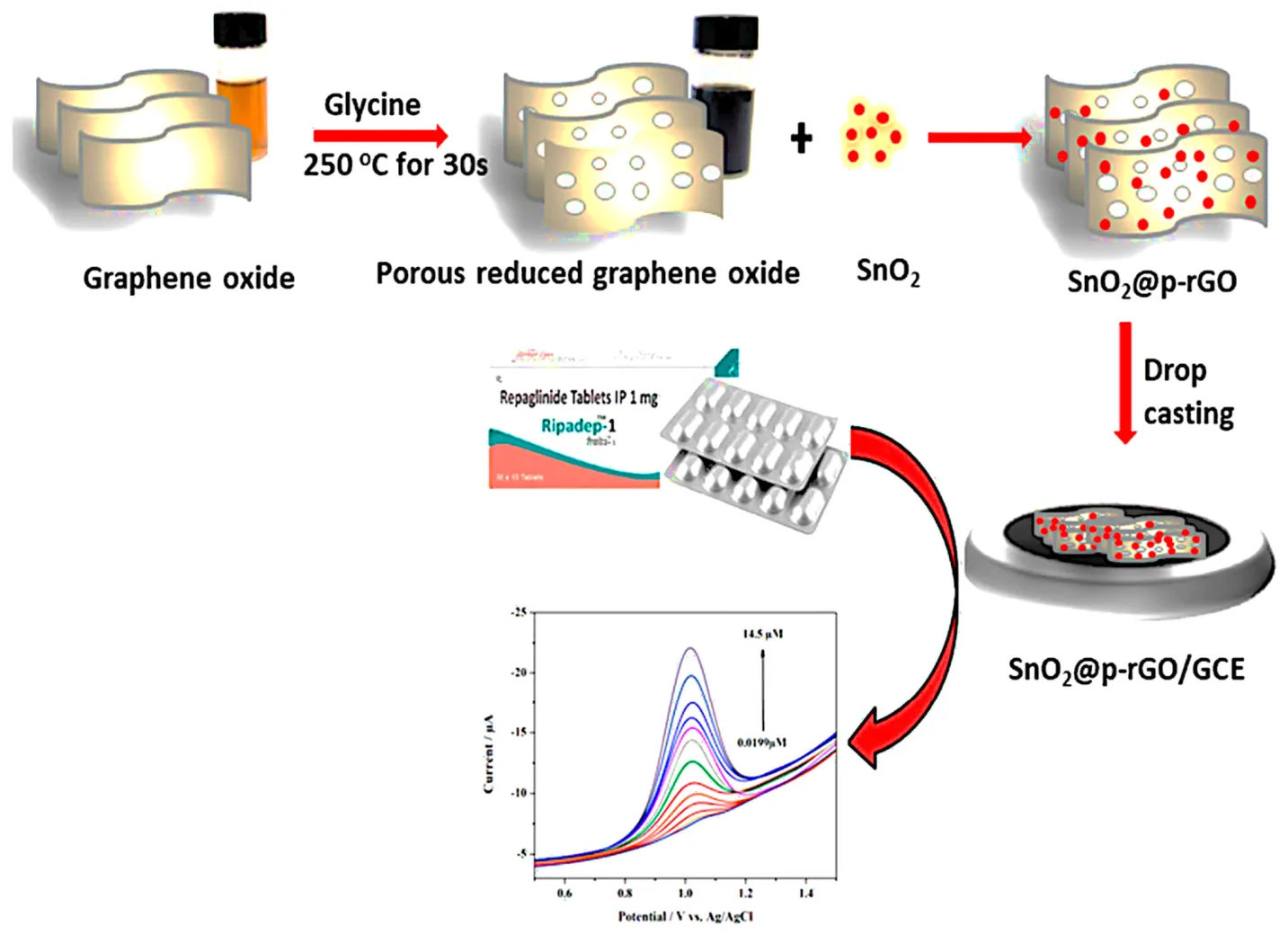
The steps involved in the fabrication of a graphene oxide–modified electrode. Reprinted from an open access source [158].

Regarding the analysis of meglitinides, both unmodified and chemically modified electrodes have been thoroughly evaluated over time. The recent literature highlights that the best analytical performance for repaglinide detection is achieved by modifying the electrode surface with a binary nanostructure consisting of tin dioxide nanoparticles anchored onto a porous reduced graphene oxide network. In this configuration, reduced graphene oxide expands the electrochemically active surface area, accelerating the electron transfer rate of the repaglinide molecule, whereas tin dioxide functions as an efficient electrocatalyst. Consequently, these two modifiers exert a clear synergistic effect that significantly enhances the sensitivity and overall analytical performance of the sensor [158], as can be seen in Figure 11.

Furthermore, this sustainable analytical approach utilizes reagents derived from plant extracts or agricultural waste, effectively minimizing the consumption of toxic or expensive chemicals. This strategy significantly enhances the ecological sustainability of the proposed electrochemical technique [158].

Specifically, the protocols developed by Mathad et al. [158] and Hosny et al. [157] highlight the advantages of integrating graphene as a primary sensor modifier alongside hematoxylin and chitosan. The growing interest in these functional materials underscores a prominent trend toward developing platforms that simultaneously expand the electrochemically active surface area and exert electrocatalytic effects. Moreover, the target antidiabetic drug, repaglinide, can also be quantified using conventional, unmodified electrodes to monitor its inherent electrochemical behavior. This approach offers a simple, rapid, and cost-effective alternative, bypassing the complex preparation steps associated with heavily modified electrode surfaces.

Additionally, investigating the electrochemical mechanism of repaglinide across a wide pH range necessitated the use of a Britton–Robinson buffer solution, allowing for precise control over the proton-dependent electron transfer steps [159].

Regarding alternative antidiabetic screening regimes, distinct modification strategies have been systematically compared to maximize detection performance. Regarding dapagliflozin, comparative investigations evaluate the performance of alternative carbon frameworks—such as a boron-doped diamond electrode (BDDE) or conventional carbon paste matrices—against heavily modified setups like a glassy carbon electrode incorporating an iron oxide-chitosan nanoparticle composite. Utilizing differential pulse voltammetry (DPV) and differential pulse adsorptive stripping voltammetry (DPAdSV), data demonstrated that incorporating biopolymers like chitosan successfully enhances the electronic response. This framework provided a significantly enhanced signal, enabling the precise detection of trace levels of the active substance when complex biological fluids and pharmaceutical formulations were used as sample matrices [160].

Canagliflozin, an oral antidiabetic drug belonging to the same therapeutic subclass, was electrochemically determined via differential pulse adsorptive stripping voltammetry using screen-printed carbon electrodes modified with iron oxide to improve sensitivity. The primary advantage of this methodology relies on the advanced conductive ink used for electrode fabrication, positioning the quantitative determination of canagliflozin within a sustainable analytical protocol [161].

These advancements underscore a prominent transition in contemporary pharmaceutical analysis toward adopting cost-effective, sustainable electrochemical methodologies that ensure high reproducibility without compromising analytical sensitivity.

**Figure 12:**
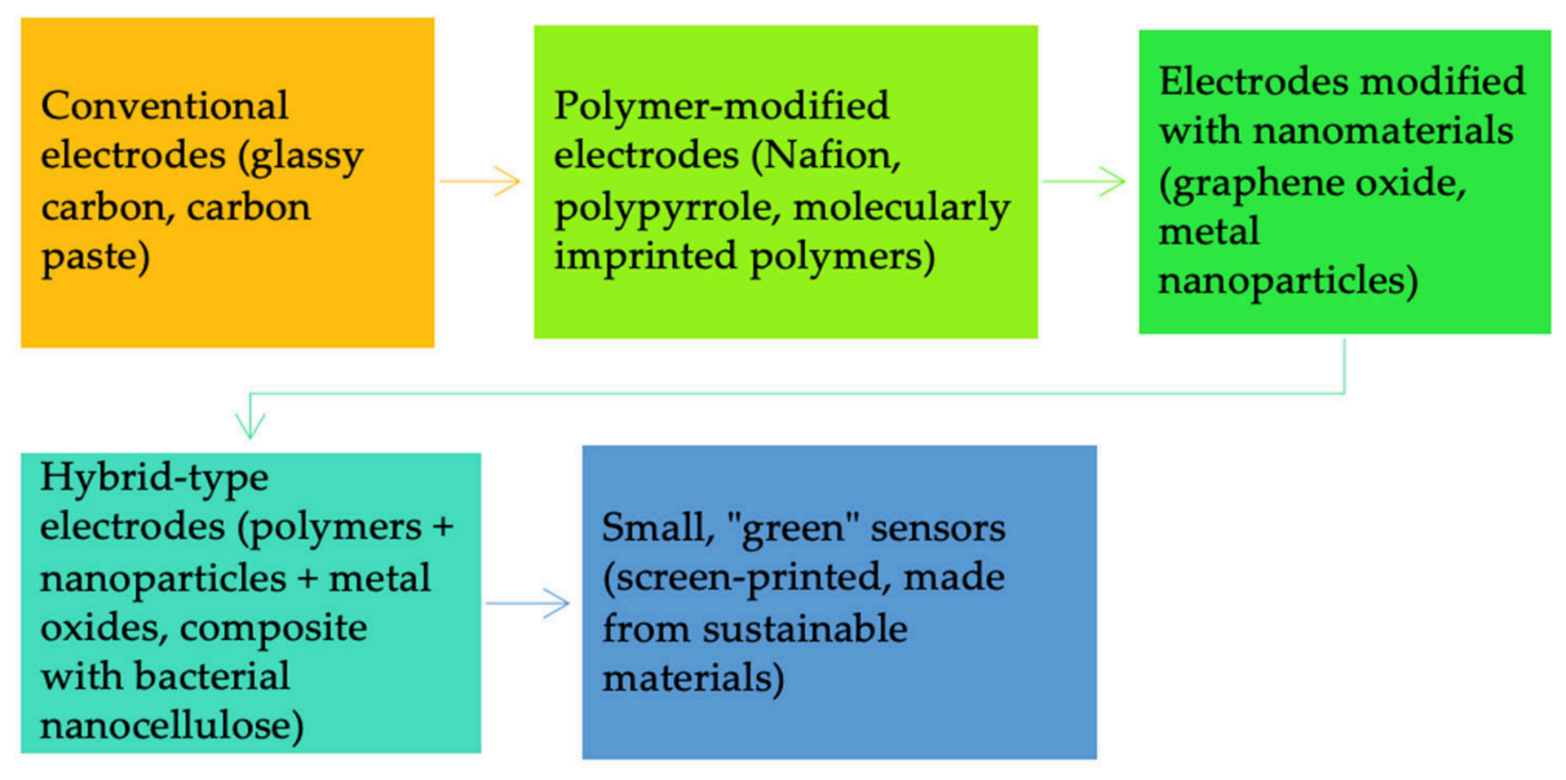
The evolution of electrodes used in quantitative determinations in electrochemistry.

As can be seen from Figure 12, beyond the possibility of achieving reliable analytical performance using conventional, unmodified electrodes, the literature has revealed a marked increase in electrochemical approaches based on modified substrates incorporating various materials, to achieve improved selectivity and sensitivity.

## 6. Future Directions

Although the literature extensively evaluates the optimization of spectrophotometric, chromatographic, and electrochemical methods for oral antidiabetic drugs, a critical gap remains regarding the fundamental coordination chemistry governing transition metal–ligand interactions. Thus, the complexes formed between oral antidiabetic agents and transition metals such as copper(II), nickel(II), zinc(II), cobalt(II), or iron(III) can support the development of these instrumental techniques, thereby enabling the quantitative determination of target pharmaceutical substances through the formation of these metal complexes.

Future research should explore chemical reaction-based signal enhancement strategies for the quantitative determination of oral antidiabetic drugs. In this context, comparative studies on the complexes formed between oral antidiabetic drugs and transition metals such as copper(II), nickel(II), iron(III), and zinc(II) may provide meaningful structural insights. Additionally, upcoming investigations should focus on optimizing complex formation conditions, systematic pH profiling and assessing the stability of the resulting products.

Given that the literature contains relevant data regarding metals such as nickel(II), cobalt(II), and copper(II), which are involved in coordination complexes with oral antidiabetic agents, future studies should expand upon these aspects by including iron(III) and zinc(II), which have been less extensively studied in this analytical framework.

In addition, DPP-4 inhibitors are recognized for their characteristic structural diversity, featuring groups such as nitrile moieties and halogen substituents, particularly fluorine (as seen in sitagliptin) within their molecules. Consequently, these structural features may influence how they interact with the transition metals, a phenomenon that warrants deeper investigation through structural and computational profiling. Additionally, the pH and the nature of the solvent used in the preparation of the complexes are among the parameters proposed for investigation in this work.

Sulfonylureas belong to another therapeutic class of oral antidiabetics and fall into a distinct structural category regarding their propensity to react with transition metals. Containing functional groups such as sulfonyl or urea moieties in their structure, they may exhibit different behavior during complexation with nickel, copper, or zinc, depending on the geometric and electronic constraints of the ligands.

Sodium-glucose cotransporter 2 inhibitors contain a carbohydrate residue and oxygen atoms derived from hydroxyl groups in their structure. Therefore, exploring the competitive complexation capabilities of metformin and sitagliptin, in which the donor centers are predominantly nitrogen, against these oxygen-rich domains represents a relevant objective for understanding multi-ligand systems in future analytical applications. Thus, beyond the study of optimizing complexation conditions between oral antidiabetic agents and transition metals, the influence of functional groups within molecular structures on complexation can also be elucidated.

Future research should also focus on UV-VIS spectrophotometry, column chromatography, and electrochemical techniques as complementary instrumental platforms. Special focus should be placed on developing advanced UV-VIS spectrophotometric methods for the analysis of the compounds of interest, both individually and within complex mixtures. In addition, atomic absorption spectroscopy (AAS) could be further investigated for indirect quantification based on the formation of metal complexes. Chromatographic methods should likewise be considered to improve selectivity and enable the reliable analysis of intricate pharmaceutical formulations.

Spectrophotometric methods are noted in the literature for their simplicity and speed, making them well-suitable for routine analytical laboratories. While not lacking in precision, they are considered among the non-destructive analytical tools, an important aspect to consider when the sample is intended to be recovered for subsequent analyses.

Although UV-VIS spectrophotometry offers key advantages such as simplicity, acceptable sensitivity and accessibility, it may be complemented by HPLC to achieve improved selectivity and more robust validation performance. In this regard, future studies should include a critical comparison between UV-VIS spectrophotometric and liquid chromatographic methods in terms of validation parameters such as sensitivity, selectivity, precision, accuracy and applicability to pharmaceutical dosage forms.

Regarding atomic absorption spectroscopy, transition metals, as components of oral antidiabetic coordination complexes, can be quantified using this method, allowing the active pharmaceutical ingredients to be analyzed quantitatively and indirectly, through these coordination structures. Within this analytical framework, the current literature is limited in its coverage of instrumental analysis techniques, being restricted mainly to glipizide and metformin.

Electrochemistry plays a significant role in the study of the complexes, finding direct application in the development of modified sensors, such as ion-selective electrodes (ISE). Thus, the complexes formed between oral antidiabetic agents and metal ions can function as electroactive chemical species within a polymer membrane, conferring upon it the ability to selectively recognize the drug substance as an analyte.

For future method development, these results obtained using these analytical techniques should be critically analyzed and interpreted in comparison with data available in the literature. Furthermore, the proposed methods must be rigorously validated in accordance with ICH guidelines to ensure international regulatory compliance.

## 7. Conclusions

From a comparative perspective, the studies compiled in this review describe the evolution of spectrophotometry from a simple, classical analytical method for the quantification of oral antidiabetic drugs into a versatile, high-throughput analytical tool. For example, while some investigations rely on conventional quantification, others have successfully enhanced the selectivity of these methods by incorporating advanced mathematical modeling, derivative spectrophotometry, or multivariate techniques. In a distinct approach, the strategic formation of metal complexes has emerged as a reliable strategy to significantly improve the analytical signal. Furthermore, research directions in recent years have increasingly focused on integrating sustainable chemistry principles, prioritizing sustainable, less toxic and environmentally friendly reagents to minimize the ecological risks associated with hazardous waste generated by traditional organic solvents. Concurrently, while the contribution of mathematical applications to enhancing analytical selectivity and precision is becoming evident, these advanced approaches are still considered niche techniques, remaining relatively inaccessible within routine pharmaceutical control.

Within the field of chromatographic analysis methods, the literature reports numerous high-performance methodologies targeting this therapeutic area. These include well-established techniques, particularly those utilizing RP-HPLC, which remain the preferred choice for routine pharmaceutical quality control of oral antidiabetics. Nevertheless, a clear technological evolution is observed from standalone RP-HPLC toward hyphenated methods, such as LC-MS/MS, UHPLC, and UPLC-DAD. These platforms are systematically selected for bioanalysis to accurately determine the pharmacokinetic, biopharmaceutical, and stability properties of the target drug substances. In contrast, there is a growing trend toward clinical and toxicological research, coupled with a decisive shift toward eco-friendly, cost-effective methods that offer enhanced analytical performance.

Regarding the literature in the field of electrochemistry, the current paradigm shift in the electroanalysis of oral antidiabetic agents is driven by the transition from conventional electrodes to chemically modified electrodes. The primary objective of this development is to achieve enhanced sensitivity and lower detection limits within pharmaceutical products and complex biological matrices.

On the other hand, bridging spectrophotometric, chromatographic, and electrochemical methods, there is an increasingly pronounced trend toward adopting sustainable methodologies that deliver analytical performance comparable to that of legacy methods using toxic and costly organic reagents. Furthermore, within the electrochemical field, the cited literature demonstrates that satisfactory reproducibility and selectivity can still be achieved using unmodified electrochemical sensors. Although this category of sensors remains uncharacterized, it represents a suitable option for future cost-effective sensor optimization, eliminating the need for expensive materials while effectively demonstrating the fundamental electrochemical properties of oral antidiabetics.

## Figures and Tables

**Table 1:** Chemical reaction-based signal enhancement methods for the quantitative determination of oral antidiabetic agents.

Pharmaceutical Agent / Sample Origin	Analytical Method	Linear Range *	LOD/LOQ (µg/mL)	Recovery / RSD (%)	Specifications	References
Gliclazide and Glibenclamide / Bulk and Tablets	Titrimetry	MB: 3–10 µg/mL MO: 2–6 µg/mL MB: 4–24 µg/mL; MO: 4–12 µg/mL;	0.89/2.68 0.43/1.43 1.12/3.38 1.07/3.24	100–100.1 99.9 / 1.04–1.12 0.91–0.92	Sample preparation: Oxidation (in situ generated bromine) and dissolution Intended application: Routine quality control of tablets	[52]
Repaglinide / Tablets	Titrimetry UV-Vis spectrophotometry	1–10 mg range 1–16 µg/ml	Not reported BCP: 0.875/2.92 BCG: 0.263/0.875	99.67–102.5 ≤2.02 98.56–103.6 98.33–104.5 / ≤2.02	Sample preparation: Oxidation (acetous perchloric) and dissolution. Ion-pair complexation (bromocresol purple and bromocresol green) and solvent extraction (dichloromethane) Intended application: Routine quality control of tablets	[53]
Pioglitazone Hydrochloride / Bulk and tablets	UV-Vis Spectrophotometry	Indirect method: 1.25–25 µg/mL	0.36/1.08	98.56–103.1 / ≤2.11	Sample preparation: Oxidation (using permanganate in acidic and basic media) and dissolution Intended application: Routine quality control of tablets	[54]
		Direct method: 1–12 µg/mL	0.23/0.69			
Saxagliptine / Bulk and pharmaceutical dosage forms	UV-Vis Spectrophotometry	6–24 µg/mL	Not Reported	99.3–100.08 / 0.13–0.799	Sample preparation: Ion-pair complexation (bromothymol blue and bromocresol green) solvent extraction (chloroform) Intended application: Routine quality control of tablets	[55]
Glibenclamide / Bulk and Pharmaceutical dosage forms	UV-Vis Spectrophotometry	2.5–12.5 µg/mL	BCP: 0.0266/0.266 BTB: 0.0142/0.142	99.19% ± 0.45 0.5831 99.59% ± 0.22 0.8941	Sample preparation: Ion-pair complexation (bromocresol purple and bromothymol blue) and solvent extraction (chloroform) Intended application: Routine quality control of tablets	[56]
Glimepiride / Bulk and Pharmaceutical dosage forms	UV-Vis spectrophotometry	0.981–9.812 µg/mL for 1 × 10^−4^ M BCG 9.812–58.874 µg/mL for 1 × 10^−3^ M BCG	0.088/0.29	98.9–102.4 / 3.0	Sample preparation: Ion-pair complexation (bromocresol green) and solvent extraction (chloroform) Intended application: Routine quality control of tablets	[57]
Metformin / Bulk and Pharmaceutical dosage forms	UV-Vis spectrophotometry	2–16 µg/ml	0.167/0.557	100.155 / <1	Sample preparation: Oxidation (with N-bromosuccinimide) and dye decolorization (Eriochrome Black-T) Intended application: Routine quality control of tablets	[58]

Abbreviations: LOD—limit of detection; LOQ—limit of quantification; RSD–relative standard deviation; MO—methyl orange; MB—methylene blue; BCP—bromocresol purple; BCG—bromocresol green; BTB—bromothymol blue. * All correlation coefficients were greater than 0.99.

**Table 2:** Methods for the quantification of metformin in pharmaceutical formulations.

Pharmaceutical Agent / Sample Origin	Method / Technique	Linear Range *	LOD/LOQ (µg/ml)	Recovery / RSD (%)	Specifications	References
Vildagliptin + Metformin / Pharmaceutical dosage forms (tablets)	UV-VIS Spectrophotometry Zero-order spectrum 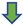 First-order spectrum	Vildagliptin: 60–100 µg/ml	Not reported	100 / Not Reported	Sample preparation: Dissolution in solvent (distilled water). Intended application: Routine quality control of tablets	[66]
		Metformin: 10–50 µg/ml				
Metformin / Pharmaceutical dosage forms and bulk powder	UV-VIS Spectrophotometry (multivariate analysis)	1–10 µg/ml	0.082/0.25	102.50 ± 0.06 / ≤0.56	Sample preparation: Dissolution in distilled water Intended application: Routine quality control of tablets	[67]
Metformin + Gliclazide 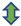 caffeine / Pharmaceutical dosage forms and bulk powder	UV-VIS spectrophotometry	Stability constants at different pH values (1.4, 2.4, 3.4, 4.4, 5.4, 6.4, 7.4) Caffeine-gliclazide: (2.57, 2.48, 35.67, 3.78, 3.75, 3.45,2.35) Caffeine-metformin: (5.48, 5.42, 3.65, 2.72, 2.45, 2.33, 2.23)	Not Reported	Not Reported	Sample preparation: Dissolution in aqueous buffer media Intended application: In vitro drug–drug interaction studies	[68]
Metformin HCl + Pioglitazone + Glimepiride / Formulation and Bulk powder	UV-VIS spectrophotometry A. Simultaneous equation method B. Derivative spectroscopy	Metformin HCl: 5–40 µg/ml	0.19/0.57 (A) 0.10/0.31 (B) 0.32/0.98 (A) 0.11/0.35 (B) 0.14/0.43 (A) 0.14/0.43 (B)	≤100 / 0.2426- 0.9821	Sample preparation: Dissolution in methanol and filtration Intended application: Routine quality control of tablets	[49]
		Pioglitazone: 5–40 µg/ml				
		Glimepiride: 5–50 µg/ml				
Metformin HCl + Canagliflozin / Pharmaceutical dosage forms (tablets)	UV-VIS Spectrophotometry (Vierordt’s equation)	Metformin HCl: 5–17.5 µg/ml Canagliflozin: 2.5–15 µg/ml	0.49/1.49 0.43/1.31	98.82 / 0.094–0.496 99.43 / 0.243–0.520	Sample preparation: Dissolution in methanol and filtration Intended application: Routine quality control of table	[69]
Glimepiride + Metformin / Pharmaceutical dosage forms	UV-VIS spectrophotometry I. First-order derivative spectroscopy II. Multicomponent mode Technique	Metformin: 1.0–5.0 µg/ml (I) 0.5–2.5 µg/ml (II) Glimepiride: 3–15 µg/ml (I) 2.0–10.0 µg/ml (II)	0.05/0.15 (I) 0.02/0.06 (II) 0.11/0.34 (I) 0.08/0.25 (II)	~100 / <1	Sample preparation: Dissolution in methanol and filtration Intended application: Routine quality control of tablets	[48]

Abbreviations: LOD—limit of detection; LOQ—limit of quantification; RSD—relative standard deviation; Rec—recovery * All correlation coefficients were greater than 0.99; 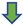: transition to first order spectrum; 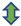: the interactions between the components.

**Table 3:** Chromatographic methods for the separation of oral antidiabetic drugs from mixtures.

Pharmaceutical Agent / Sample Origin	Mobile Phase	Stationary Phase	Detection Mode	Results * Linearity, LOD/LOQ (µg/mL)	Specifications	References
Saxagliptin + Dapagliflozin / Human plasma	0.1% orthophosphoric acid: acetonitrile (50:50, *v*/*v*) pH = 5.0	Eclipse XDB-C18	UV detection	Saxagliptin: 0.01–0.5 Dapagliflozin: 0.05–2 LOD, LOQ have not been reported	Specs: PP (ACN); RT: 10.0 min; Rec/RSD: 71.6–88.07%/<2%; ME/Stab: Neg./Stable for 24 h at room temperature and 37 days at −28 °C; App: PK and BE	[100]
Linagliptin + Dapagliflozin + Degradation products / Synthetic mixture	Gradient of 0.1% formic acid and acetonitrile	Eclipse XDB-C18	PDA for quantification / LC-MS/MS for degradant analysis	Linagliptin: 25–75 4/12 Dapagliflozin: 50–150 5/15	Specs: Forced degradation, extraction with diluent, and filtration; RT: 12.0 min; Rec/RSD: 71.6–88.07%/<2%; ME/Stab: N/A; App: Stability-indicating and degradant analysis of tablets	[101]
Dapagliflozin + Saxagliptin / Human plasma	0.1% formic acid: acetonitrile (25:75, *v*/*v*)	Hypersil C18	UV detection	Linearity: Dapagliflozin: 50–1050 ng/mL Saxagliptin: 75–1200 ng/mL LOD, LOQ have not been reported	Specs: PP (ACN); RT: ~10.0 min; Rec/RSD: ~100%/1.00–3.04%; ME/Stab: Neg./Stable after three freeze–thaw cycles and benchtop stability tests; App: PK and BE studies	[102]
Dapagliflozin + Vildagliptin / Pharmaceutical dosage forms	Gradient of orthophosphoric acid and acetonitrile, pH = 5.0	Agilent C18 column	UV detection	Linearity: Dapagliflozin: 0.032–10 Vildagliptin: 0.32–100 LOD, LOQ have not been reported	Specs: Dissolution in solvent and filtration; RT: ~10.0 min; Rec/RSD: 98–102%/<2% ME/Stab: N/A; App: Routine quality control of tablets	[103]
Dapagliflozin + Saxagliptin / Bulk powder	Methanol: water (45:55, *v*/*v*)	Intersil ODS-C18	UV detection	Dapagliflozin: 0–70 ppm 0.56/1.69 Saxagliptin: 0–70 ppm 0.57/1.74	Specs: Dissolution in solvent and filtration; RT: ~12.0 min; Rec/RSD: ~99.61–100.21%/0.031%(DAP), 0.036 {SAX)%; ME/Stab: N/A; App: Routine quality control of tablets	[104]
Teneligliptin+ Metformin / Fixed-dose combination	Phosphate buffer 50 mM (pH 3.5): acetonitrile = 65:35, *v*/*v*	Eclipse plus C18	UV detection	Teneligliptin: 20–100 1.29/3.93 Metformin: 20–100 2.87/8.71	Specs: Forced degradation, extraction with solvent, and filtration; RT: ~15.0 min; Rec/RSD: ~98.39–101.87%; <2%. ME/Stab: N/A; App: Stability-indicating and routine quality control of tablets	[105]
Ertugliflozin+ Metformin / Tablets	Phosphate buffer 0,01N: acetonitrile (60:40, *v*/*v*)	Ascentis C18	UV detection	Ertugliflozin: 0.375–2.25 0.02/0.05 Metformin: 25–150 0.75/2.28	Specs: Dissolution in solvent and filtration; RT: not reported; Rec/RSD: 99.75%(MET), 100.13% (ERT); 1.1%(MET), 0.7 %(ERT). ME/Stab: N/A; App: Routine quality control and degradation studies of tablets	[106]
Metformin+ Saxagliptin+ Dapagliflozin / Pharmaceutical dosage forms	KH_2_PO_4_ 20 mM: acetonitrile (30:70, *v*/*v*), pH 3.5 adjusted with OPA	Prontosil C18 column	UV detection	Metformin: 5–25 g/ml 0.5/1.5 Saxagliptin: 5–25 g/ml 0.012/0.035 Dapagliflozin: 0.1–0.5 g/ml 0.018/0.05	Specs: Dissolution in solvent and filtration; RT: ~15.00 min; Rec/RSD: 95.11–98.97%/<2%. ME/Stab: N/A; App: Routine quality control of tablets	[107]
Vildagliptin +Metformin / Pharmaceutical dosage forms	Acetonitrile: methanol: water (15:60:25, *v*/*v*)	Hypersil C18	UV detection	Metformin: 50–150% 0.617/1.87 Vildagliptin: 50–150% 0.154/0.468	Specs: Dissolution in solvent and filtration; RT: nor reported; Rec/RSD: ~100.00%/<2%. ME/Stab: N/A; App: Routine quality control of tablets	[108]

Abbreviations: LOD—limit of detection; LOQ—limit of quantification; Specs—method specifications; PP—protein precipitation; ACN—acetonitrile; RT—chromatographic run time; Rec/RSD—recovery/relative standard deviation; ME/Stab—matrix effects/sample stability; App—intended application; PK—pharmacokinetics; BE—bioequivalence; SAX—saxagliptin; DAP—dapagliflozin; MET—metformin; ERT—ertugliflozin; Neg.—negligible; N/A—not applicable; OPA—orthophosphoric acid. * All correlation coefficients were greater than 0.99.

**Table 4:** Analytical methods for the quantification of thiazolidinediones and related compounds in various samples.

Pharmaceutical Agent / Sample Origin	Mobile Phase	Stationary Phase	Detection Mode	Detection Wavelenght (or Mass Spectrometry Conditions)	Results * Linearity, LOD, LOQ (μg/mL)	Specifications	References
Saroglitazar+ Linagliptin+ Teneligliptin / Bulk powder	Phosphate buffer (pH 7.0): acetonitrile (different ratio)	Phenomenex Luna C-18 Column	UV Detection	243 nm	Saroglitazar: 0.1–0.5 0.037/0.188 Linagliptin: 0.1–0.5 0.014/0.074 Teneligliptin: 0.1–0.5 0.096/0.482	Specs: Dissolution in solvent and filtration; RT: 12.0 min; Rec/RSD: ~100.00% /<2%. ME/Stab: N/A; App: Routine QC	[109]
Pioglitazone hydrochloride / Pharmaceutical dosage forms	5 mM solution of ammonium Acetate: acetonitrile	Inertsil ODS-3 C18 column	High-resolution mass spectrometry (HRMS)	Spray voltage: 3.8 kV Capillary temperature:320 °C Vaporizer temperature: 250 °C sheath Gas, auxiliary gas, and S-lens RF levels: 40, 10, 50 arbitrary units	•High-resolution mass accuracy: <5 ppm •Structural elucidation via fragmentation patterns •Identification of potential genotoxic alerts.	Specs: Dissolution in solvent and filtration; RT: not reported; Rec/RSD: N/A. ME/Stab: N/A; App: Impurity profiling and structural identification in formulation.	[110]
Rosiglitazone / Human plasma	0.01M dibasic potassium hydrogen phosphate (pH 6.5): 0.01M acetonitrile (65:35, *v*/*v*)	Nova-pak C18 cartridge	Multi- wave length fluorescence detector	247 nm	5–800 ng/ml 2.5 ng/ml/5 ng/ml	Specs: LLE with methylene chloride and hexane after acidification with 1 M HCl; RT: 9.00 min; Rec/RSD: 91–99%/<7.7%. ME/Stab: Neg./Stable in human plasma under various storage conditions App: Bioanalytical validation and human plasma drug monitoring	[111]
Pioglitazone / Human plasma	Acetonitrile ammonium acetate 0.1 M (41:59, *v*/*v*), pH =4.10	C18 Column	UV Detection	269 nm	0.055–2.0 25 ng/mL/ 55 ng/mL	Specs: PP and extraction with acetonitrile; RT: N/A; Rec/RSD: >79%/<3%. ME/Stab: Neg/Stable in human plasma during short-term and freeze–thaw validation cycles App: PK studies in human volunteers	[112]
Pioglitazone / Human plasma	50 mM phosphate buffer (pH 4.53): acetonitrile (55:45, *v*/*v*)	Inersil ODS-3 C18 column	UV Detection	229 nm	Linearity, LOD, LOQ have not been reported.	Specs: Protein precipitation and extraction with acetonitrile; RT: N/A; Rec/RSD: N/A ME/Stab: Neg./ Stable under clinical validation storage conditions App: Human pharmacokinetic and bioequivalence studies	[113]
Pioglitazone synthetic analogue / Human and rat plasma	Methanol: water (30: 70, *v*/*v*)	C18 Column	UV detection	280 nm	0.3–50.0 N/A/2	Specs: Protein precipitation with methanol RT: 7.0 min; Rec/RSD: 89.94–108.06%/<12%. ME/Stab: Neg./ Stable under clinical validation storage conditions App: Pharmacokinetic studies in rat plasma	[114]

Abbreviations: RSD—relative standard deviation; LOD—limit of detection; LOQ—limit of quantification; Specs—method specifications; PP—protein precipitation; LLE—liquid–liquid extraction; RT—chromatographic run time; Rec/RSD—recovery/relative standard deviation; ME/Stab—matrix effects/sample stability; App—intended application; PK—pharmacokinetics; QC—quality control; Neg.—negligible; N/A—not reported. *All correlation coefficients were greater than 0.99.

## Data Availability

No new data were created or analyzed in this study. Data sharing is not applicable in this article.

## Supplementary Materials

The following supporting information can be downloaded at: https://www.mdpi.com/article/10.3390/molecules31162915/s1. Literature search strategy and methodology.
